# The NSP5, ORF6 and NSP13 of SARS‐CoV‐2 Cooperate to Modulate Inflammatory Cell Death Activation

**DOI:** 10.1002/advs.202503977

**Published:** 2025-08-14

**Authors:** Huan Wang, Mengdi Liang, Jing Zhang, Hua Tong, Fenfen Zhang, Ying Liu, Pui Wang, Mengmeng Chang, Fei Han, Siwen Liu, Yongping Lin, Wenjun Song, Rajendra Karki, Peihui Wang, Honglin Chen, Yang Liu, Min Zheng

**Affiliations:** ^1^ Institute of infectious diseases Shenzhen Bay Laboratory Shenzhen Guangdong 518132 China; ^2^ Guangdong Provincial Key Laboratory of Infection Immunity and Inflammation Shenzhen Guangdong 518060 China; ^3^ Key Laboratory for Experimental Teratology of Ministry of Education and advanced Medical Research Institute Meili Lake Translational Research Park Cheeloo College of Medicine Shandong University Jinan 250012 China; ^4^ Department of Microbiology and State Key Laboratory for Emerging Infectious Diseases the University of Hong Kong Hong Kong 999077 China; ^5^ Department of Preventive Veterinary Medicine Guangxi University Nan Ning 530004 China; ^6^ Department of Laboratory Medicine the First Affiliated Hospital of Guangzhou Medical University Guangzhou 510030 China; ^7^ KingMed School of Laboratory Medicine Guangzhou Medical University Guangzhou 510182 China; ^8^ Guangzhou Laboratory Guangzhou International Bio Island Guangzhou 510005 China; ^9^ Department of Biological Sciences College of Natural Science Seoul National University Seoul 08826 South Korea

**Keywords:** caspase‐8, necroptosis, NSP13, PANoptosis, RIPK3, SARS‐CoV‐2, ZBP1

## Abstract

Programmed cell death is a pivotal mechanism of cell‐autonomous immune defense against viral infections. Recent studies indicate that both blocking and promoting cell death negatively affect coronavirus replication, implying that coronaviruses may fine‐tune cell death pathways to optimize their propagation. However, the mechanisms underlying this remain poorly understood. Here, it is verified that coronaviruses induce the formation of a Z‐DNA‐binding protein 1 (ZBP1)‐initiated cell death complex involving ZBP1, Z‐RNA, receptor‐interacting serine/threonine‐protein kinase 3 (RIPK3), and caspase‐8, thereby triggering apoptosis, pyroptosis, and necroptosis in human bronchial epithelial cells. To impede the activation of apoptosis and pyroptosis, NSP5 and ORF6 of SARS‐CoV‐2 concurrently inhibit caspase‐8 activity by targeting its large and small subunits, respectively. Additionally, NSP13, the viral helicase, interacts with RIPK3 to impair its binding to ZBP1, thus suppressing ZBP1‐initiated necroptosis. This inhibitory effect on cell death is likely conserved across β‐coronaviruses. Furthermore, co‐infection of influenza A virus and SARS‐CoV‐2 is demonstrated to exacerbate disease severity, although the mechanisms remain unclear. These findings suggest that β‐coronavirus‐induced inhibition of cell death enhances influenza A virus replication and worsens inflammation during their co‐infection, ultimately increasing mortality in mice. This research provides valuable insights into the regulation of coronavirus‐induced cell death, offering potential therapeutic strategies for combating highly pathogenic coronavirus infections.

## Graphical abstract

ZBP1 is capable of initiating a large cell death complex to induce programmed cell death during SARS‐CoV‐2 infection. However, SARS‐CoV‐2 can inhibit the activation of ZBP1‐mediated cell death by targeting key components of this complex. This suppression of ZBP1‐mediated cell death may account for the increased mortality observed in patients co‐infected with influenza A virus and SARS‐CoV‐2.

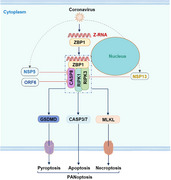

## 1 Introduction

Coronaviruses belong to the family Coronaviridae, which consists of four genera: α‐coronavirus, β‐coronavirus, γ‐coronavirus, and δ‐coronavirus. Highly pathogenic coronaviruses have posed significant threats to global health. Over the past two decades, three such viruses─severe acute respiratory syndrome coronavirus (SARS‐CoV), Middle East respiratory syndrome coronavirus, and SARS‐CoV‐2 (SARS2), all classified within the β‐coronavirus genus─have emerged, causing substantial mortality and morbidity.^[^ ^1^ ^]^ The coronavirus disease 2019 (COVID‐19), caused by SARS2, has resulted in over 6 million deaths worldwide since 2019.^[^ ^2^ ^]^ Common complications in severe cases of these infections include acute respiratory distress syndrome and respiratory failure.^[^ ^3^, ^4^ ^]^ It has been suggested that dysregulated cell death and inflammation are primary contributors to lung damage during highly pathogenic coronavirus infections.^[^ ^1^, ^5^, ^6^, ^7^ ^]^ However, the mechanisms underlying the regulatory processes are still largely unexplored.

Innate immunity serves as the first line of host defense against pathogen infections, with programmed cell death and inflammation playing pivotal roles in this process. Programmed cell death can occur in either an immunologically silent (non‐lytic) or inflammatory (lytic) manner.^[^ ^8^ ^]^ Apoptosis is a form of non‐lytic cell death, which can be subdivided into intrinsic and extrinsic apoptosis. Intrinsic apoptosis involves the activation of caspase‐9, which subsequently activates the executioner caspases, caspase‐3 and ‐7, to induce cell death.^[^ ^8^ ^]^ In contrast, extrinsic apoptosis is mediated by caspase‐8, which then activates caspase‐3 and ‐7.^[^ ^8^ ^]^ Both pyroptosis and necroptosis are classified as inflammatory cell death. Pyroptosis is mediated by gasdermin family members, whose N‐terminal domain can be released by specific proteases, allowing for oligomerization and pore formation in the cell membrane, ultimately causing cell rupture.^[^ ^9^ ^]^ Necroptosis, on the other hand, is driven by mixed lineage kinase domain‐like pseudokinase (MLKL), whose phosphorylation by receptor‐interacting serine/threonine‐protein kinase 3 (RIPK3) triggers its oligomerization and pore formation in the cell membrane, leading to lytic cell death.^[^ ^10^, ^11^ ^]^ Recently, a novel form of inflammatory cell death, termed PANoptosis, has been identified in studies examining the cross‐talk between these three cell death pathways.^[^ ^12^, ^13^ ^]^ PANoptosis is activated after specific innate sensors recognize danger signals and subsequently recruit RIPK3, receptor‐interacting serine/threonine‐protein kinase 1 (RIPK1), and caspase‐8 to assemble a cell death complex, known as the PANoptosome. During the activation of PANoptosis, markers of apoptosis, pyroptosis, and necroptosis can be concurrently detected within the same cell population.

Z‐DNA‐binding protein 1 (ZBP1) is one of the innate sensors that can trigger PANoptosis upon the detection of Z‐nucleic acids.^[^ ^13^, ^14^ ^]^ Influenza A virus (IAV) is recognized by ZBP1, and its defective genomes can form Z‐RNA, thereby activating ZBP1. This leads to the recruitment of RIPK3, RIPK1, and caspase‐8, culminating in the induction of apoptosis, pyroptosis, and necroptosis, collectively referred to as PANoptosis.^[^ ^15^, ^16^, ^17^, ^18^ ^]^ The absence of ZBP1 abolishes IAV‐induced cell death. Coronaviruses, including SARS2, have also been shown to induce ZBP1‐dependent PANoptosis through the recruitment of RIPK3 and caspase‐8 in macrophages.^[^ ^6^, ^19^ ^]^ Furthermore, the deletion of the Z‐nucleic acid sensing domain of ZBP1 inhibits coronavirus‐induced cell death, suggesting that Z‐RNA or Z‐DNA is generated during coronavirus infection. Further investigations have indicated that Z‐RNA is produced during SARS2 replication and may act as a ligand to activate ZBP1 in lung epithelial cells.^[^ ^7^, ^20^, ^21^, ^22^ ^]^ Additionally, bioinformatics analyses have revealed that the NSP13 protein of coronaviruses contains a RIP homotypic interaction motif (RHIM) domain, and its overexpression has been shown to promote ZBP1‐mediated cell death in bat cells.^[^ ^20^, ^23^ ^]^ However, the precise regulatory mechanisms of ZBP1‐mediated cell death during coronavirus infection remain largely unexplored.

Programmed cell death, including apoptosis, plays a critical role in eliminating infected cells. However, blocking apoptosis has been associated with reduced coronavirus replication,^[^ ^1^, ^24^ ^]^ implying that coronaviruses may exploit apoptotic pathways to promote their proliferation. As obligate intracellular pathogens, coronaviruses rely on host cell viability for replication. Notably, increased apoptosis has been shown to limit SARS2 replication,^[^ ^25^ ^]^ indicating that coronaviruses likely modulate programmed cell death to optimize their replication process. Although extensive research has been conducted on how the host triggers ZBP1‐dependent cell death in response to coronavirus infection, the mechanisms by which the viruses regulate this process remain insufficiently understood.

In the present study, we demonstrated that coronavirus infection induces the assembly of a ZBP1‐initiated cell death complex through the coordinated recruitment of Z‐RNA, ZBP1, RIPK3, and caspase‐8, leading to the activation of PANoptosis in human bronchial epithelial cells. Our investigation revealed the indispensable roles of both Zα2 and RHIM1 domains of ZBP1 in the assembly of this complex following coronavirus infection. Deletion of either domain disrupts complex formation and impairs cell death induction. Additionally, we examined the involvement of caspase‐8 catalytic activity in this process and found it to be crucial for the induction of both apoptosis and pyroptosis during coronavirus infection in human bronchial epithelial cells. Notably, our findings identified NSP5, the main protease of SARS2, and ORF6, an accessory protein of SARS2, as key regulators of caspase‐8 activity. NSP5 specifically targets the large subunit of caspase‐8, while ORF6 interacts with the small subunit, thereby suppressing caspase‐8 activation. Moreover, we observed that NSP13, the helicase of SARS2, disrupts the interaction between RIPK3 and ZBP1, thereby preventing RIPK3 recruitment into the ZBP1‐initiated cell death complex and inhibiting necroptosis activation. Taken together, our results demonstrate that coronaviruses can inhibit ZBP1‐mediated cell death via targeting caspase‐8 and RIPK3 simultaneously. Importantly, this inhibition of cell death enhances the replication of IAV and amplifies inflammatory responses in the lungs of mice co‐infected with IAV and coronavirus, resulting in increased mortality. These findings provide valuable insights into the intricate mechanisms by which coronaviruses regulate ZBP1‐mediated cell death during infection and offer new perspectives on the pathogenesis of highly pathogenic coronaviruses.

## 2 Results

### 2.1 Z‐RNA Forms Specks with ZBP1 during Coronavirus Infection

Coronavirus infection has been shown to be recognized by ZBP1, triggering apoptosis, pyroptosis, and necroptosis via caspase‐8 and RIPK3 in mouse macrophages.^[^ ^6^ ^]^ However, the mechanisms underlying coronavirus‐induced cell death in other cell types, particularly lung epithelial cells, remain inadequately understood. To investigate this, we infected Beas‐2B, a normal human bronchial epithelial cell line, with human β‐coronavirus OC43 (hCoV‐OC43). Our findings demonstrated that hCoV‐OC43 infection in Beas‐2B cells induced the expression of ZBP1 (Figure S1A, Supporting Information). Furthermore, other key components involved in programmed cell death activation, such as RIPK1, RIPK3, caspase‐1, gasdermin E (GSDME), gasdermin D (GSDMD), and ASC, were constitutively expressed in this cell line (Figure S1A, Supporting Information). While NLRP3 expression was detected at low levels, it did not increase following hCoV‐OC43 infection (Figure S1A, Supporting Information). Consistent with previous studies,^[^ ^6^, ^7^ ^]^ hCoV‐OC43 infection‐induced cell death was almost abolished in ZBP1‐deficient Beas‐2B cells (Figure S1B,C, Supporting Information). Meanwhile, the constitutive expression of ZBP1 in Beas‐2B (Beas‐2B‐ZBP1) cells significantly enhanced hCoV‐OC43‐induced cell death (Figure S1D, Supporting Information). This enhancement was characterized by increased pyroptosis, as evidenced by the p30 cleaved fragments of GSDMD and GSDME, apoptosis, indicated by caspase‐8, ‐7, and ‐3 activation, and necroptosis, marked by the presence of phosphorylated MLKL in Beas‐2B‐ZBP1 cells compared to controls (Figure S1E–G, Supporting Information). Taken together, these findings suggest that β‐coronavirus infection can activate ZBP1‐mediated inflammatory cell death in human bronchial epithelial cells.

**Figure 1:**
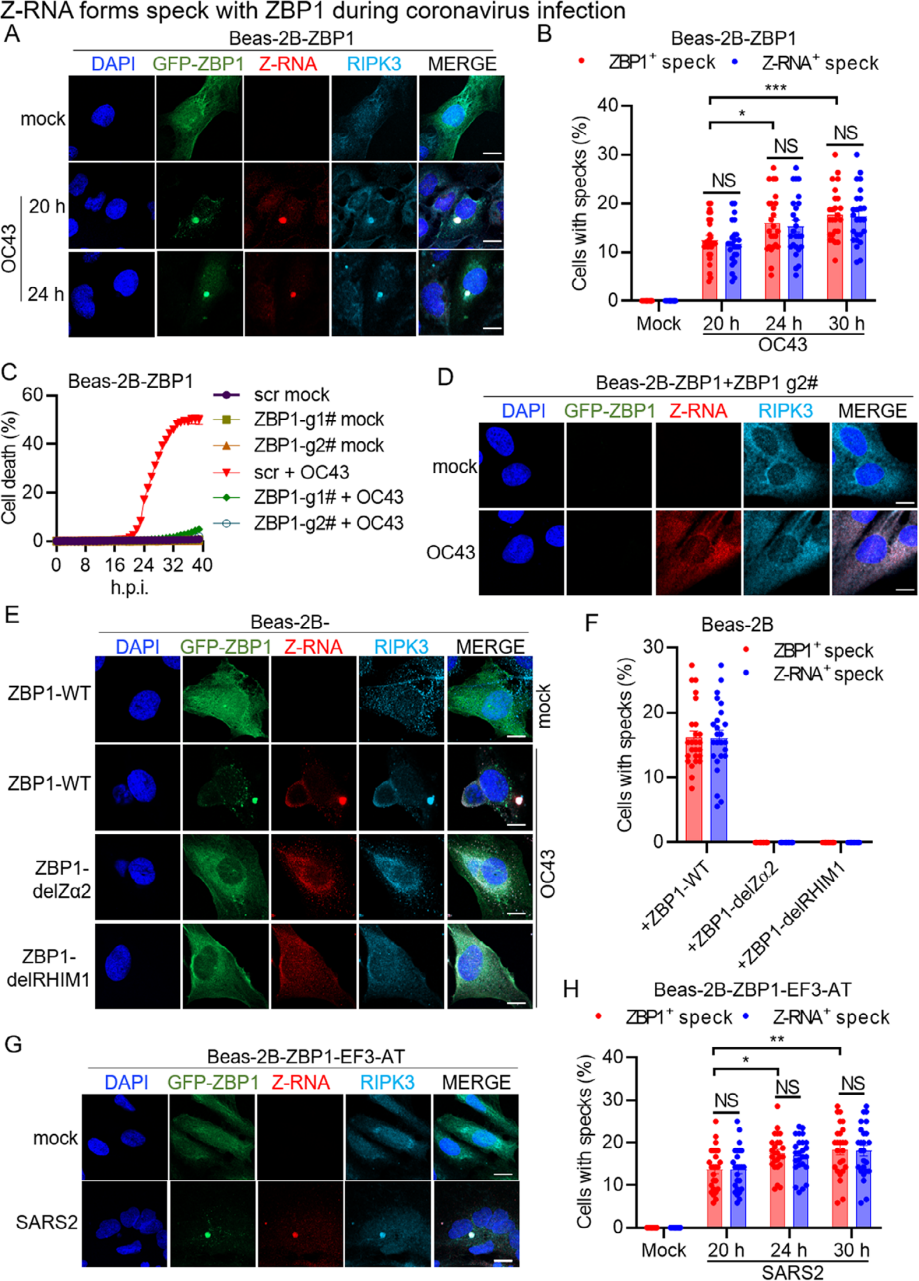
Z‐RNA forms a speck with ZBP1 during coronavirus infection. A) Confocal images of Beas‐2B‐ZBP1 cells infected with hCoV‐OC43 (OC43) at the indicated time points. Scale bar, 10 µm. B) Quantification of the percentage of cells with ZBP1^+^ or Z‐RNA^+^ specks in Beas‐2B‐ZBP1 cells infected with OC43 at the indicated time points. C) Real‐time analysis of cell death in Beas‐2B‐ZBP1 cells following CRISPR‐directed deletion of ZBP1 in response to OC43 infection. D) Confocal images of Beas‐2B‐ZBP1 cells following CRISPR‐directed deletion of ZBP1 in the presence of OC43. Scale bar, 10 µm. E) Confocal images of Beas‐2B cells stably expressing wild type (WT) ZBP1 (ZBP1‐WT), ZBP1 deleting Zα2 (ZBP1‐del Zα2), or ZBP1 deleting RHIM1 (ZBP1‐delRHIM1) infected with OC43 for 24 h. Scale bar, 10 µm. F) Quantification of the percentage of cells with ZBP1^+^ or Z‐RNA^+^ specks in E. G) Confocal images of Beas‐2B‐ZBP1‐E‐ORF3‐ACE2‐TMPRSS2 (Beas‐2B‐ZBP1‐EF3‐AT) cells infected with SARS‐CoV‐2 (SARS2) for 24 h. Scale bar, 10 µm. H) Quantification of the percentage of cells with ZBP1^+^ or Z‐RNA^+^ specks in Beas‐2B‐ZBP1‐EF3‐AT cells infected with SARS2 at the indicated time points. NS, not significant; *P < 0.05; **P < 0.01; ***P < 0.001. Analysis was performed using two‐way ANOVA (B,H). Data are shown as mean ± SEM (*n* = 25) (B, F, and H) or (*n* = 4) (C). Data are representative of three independent experiments.

β‐coronavirus‐induced cell death has been proposed to involve the formation of a ZBP1‐initiated cell death complex.^[^ ^6^, ^19^ ^]^ However, how this complex is assembled during β‐coronavirus infection remains insufficiently explored. To investigate its formation, we utilized immunofluorescence microscopy. Our results showed that hCoV‐OC43 infection led to the accumulation of ZBP1 specks in the cytoplasm (**Figure** **1**A,B). As expected, RIPK3 was recruited to these ZBP1 specks (Figure 1A). Consistent with previous findings,^[^ ^7^ ^]^ Z‐RNAs were detected following β‐coronavirus infection (Figure 1A). Notably, these Z‐RNAs also formed specks that co‐localized with ZBP1 (Figure 1A,B). These findings suggest that β‐coronavirus infection induces the assembly of ZBP1‐initiated cell death complex, with Z‐RNA representing a critical component.

To verify the essential role of ZBP1 in this complex assembly, we knocked out ZBP1 in Beas‐2B‐ZBP1 cells (Figure S1H, Supporting Information). Following hCoV‐OC43 infection, cell death was nearly abolished in ZBP1‐deficient cells (Figure 1C), further supporting the essential role of ZBP1 in driving β‐coronavirus‐induced cell death in human bronchial epithelial cells. While Z‐RNA expression remained unaffected, no Z‐RNA or RIPK3 specks were detected in the absence of ZBP1 following hCoV‐OC43 infection (Figure 1D). Previous studies have demonstrated that ZBP1 activation relies on its Zα2 and RHIM1 domains.^[^ ^7^, ^15^, ^26^, ^27^, ^28^, ^29^ ^]^ To test whether these domains are required for the ZBP1‐initiated cell death complex, we established two Beas‐2B cell lines expressing ZBP1 lacking either the Zα2 (ZBP1‐delZα2) or RHIM1 (ZBP1‐delRHIM1) domain (Figure S1I, Supporting Information). In line with previous reports,^[^ ^15^, ^26^, ^27^, ^28^, ^29^ ^]^ deletion of either domain abrogated ZBP1‐mediated cell death following hCoV‐OC43 infection (Figure S1J, Supporting Information). Importantly, the absence of either domain prevented the formation of ZBP1, Z‐RNA, and RIPK3 specks (Figure 1E,F). To further validate the assembly of this complex during β‐coronavirus infection, we utilized a *trans*‐complementation system for SARS2 infection, as previously described.^[^ ^30^ ^]^ This system allowed us to infect SARS2, which lacks the ORF3 and E genes, in a BSL‐2 lab using a cell line that heterogeneously expressed the ORF3 and E proteins of SARS2. Then, a Beas‐2B cell line (Beas‐2B‐ZBP1‐EF3‐AT) stably expressing the ORF3 and E, as well as ACE2 and TMPRSS2, the entry receptors for SARS2, was established for cell death analysis via transducing these genes into the Beas‐2B‐ZBP1 cells (Figure S1K, Supporting Information). SARS2 replication in this cell line was validated by the expression of NSP5 and NP proteins (Figure S1L, Supporting Information). Similar to hCoV‐OC43, SARS2 infection induced the formation of ZBP1 specks, with co‐localization of Z‐RNAs and RIPK3 (Figure 1G,H). Collectively, these data provide compelling evidence that β‐coronavirus infection triggers the assembly of the ZBP1‐initiated cell death complex, involving Z‐RNAs, and that both the Zα2 and RHIM1 domains of ZBP1 are indispensable in this process.

### 2.2 The Catalytic Activity of Caspase‐8 is Crucial for Coronavirus Infection‐Induced Cell Death in Human Bronchial Epithelial Cells

**Figure 2:**
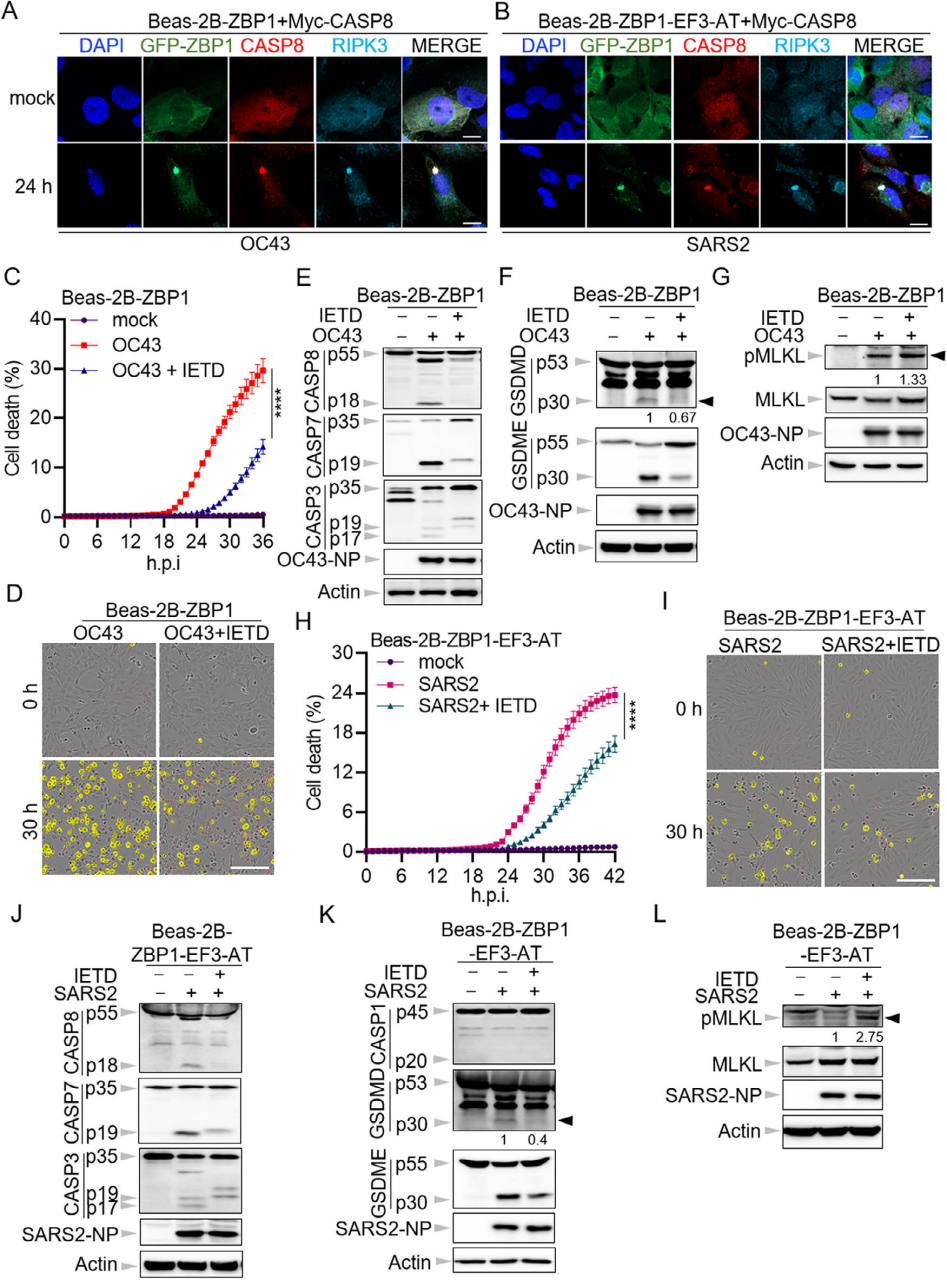
The catalytic activity of caspase‐8 is crucial for coronavirus infection‐induced cell death in human bronchial epithelial cells. A,B) Confocal images of caspase‐8 (CASP8) knock‐out Beas‐2B‐ZBP1 cells overexpressing Myc‐CASP8 (Beas‐2B‐ZBP1+Myc‐CASP‐8) after infection with hCoV‐OC43 (OC43) (A) and CASP8 knock‐out Beas‐2B‐ZBP1‐E‐ORF3‐ACE2‐TMPRSS2 cells overexpressing Myc‐CASP8 (Beas‐2B‐ZBP1‐EF3‐AT + Myc‐CASP8) after infection with SARS‐CoV‐2 (SARS2) (B) for 24 h. Scale bar, 10 µm. C) Real‐time analysis of cell death in OC43‐infected Beas‐2B‐ZBP1 cells with or without Z‐IETD‐FMK (IETD). D) Representative images of cell death at the indicated time points in C. The *yellow* in the images denotes the dead cells counted during the analysis. Scale bar, 200 µm. E–G) Immunoblot analysis of pro‐ and cleaved forms of CASP8, caspase‐7 (CASP7), ‐3 (CASP3), and OC43 nucleocapsid protein (NP) (E), pro‐ and cleaved forms of gasdermin D (GSDMD), gasdermin E (GSDME), and OC43 NP (F), phosphorylated mixed lineage kinase domain‐like protein (pMLKL), total MLKL, and OC43 NP (G) in (D). Actin is used as the internal control. H) Real‐time analysis of cell death in SARS2 infected Beas‐2B‐ZBP1‐EF3‐AT cells with or without IETD. I) Representative images of cell death at the indicated time points in H. The *yellow* in the images denotes the dead cells counted during the analysis. Scale bar, 200 µm. J–L) Immunoblot analysis of pro‐ and cleaved forms of CASP8, CASP7, CASP3, and SARS2 NP (J), pro‐ and cleaved forms of caspase‐1 (CASP1), GSDMD and GSDME, and SARS2 NP (K), pMLKL, total MLKL, and SARS2 NP (L) in I. Actin is used as the internal control. ****P < 0.0001. Analysis was performed using two‐way ANOVA (C,H). Data are shown as mean ± SEM (*n* = 3–4) (C,H). Data are representative of three independent experiments.

Caspase‐8 has been identified as a key component of the ZBP1‐initiated cell death complex, playing a key role in mediating apoptosis following influenza virus infection.^[^ ^12^, ^15^, ^17^, ^31^ ^]^ To investigate its involvement during β‐coronavirus infection in human bronchial epithelial cells, we initially assessed the recruitment of caspase‐8 to the cell death complex via the co‐immunoprecipitation assay following infection with hCoV‐OC43 in wild‐type Beas‐2B cells. It was demonstrated that both RIPK3 and caspase‐8 can be immunoprecipitated by ZBP1 following hCoV‐OC43 infection (Figure S2A, Supporting Information), suggesting that caspase‐8 is able to interact with ZBP1 during β‐coronavirus infection in human bronchial epithelial cells. To further validate this result, immunofluorescence microscopy was utilized to visualize the localization of caspase‐8 during the infection. Due to the lack of suitable caspase‐8 antibodies for confocal microscopy, we generated two cell lines stably expressing Myc‐tagged caspase‐8 in caspase‐8 deficient Beas‐2B‐ZBP1 (Beas‐2B‐ZBP1 + Myc‐CASP8) and Beas‐2B‐ZBP1‐EF3‐AT (Beas‐2B‐ZBP1‐EF3‐AT + Myc‐CASP8) cells (Figure S2B,C, Supporting Information). Following hCoV‐OC43 and SARS2 infection, we observed the emergence of caspase‐8 specks, which co‐localized with ZBP1 and RIPK3 (**Figure** **2**A,B). These findings support the notion that caspase‐8 is a critical component of the ZBP1‐initiated cell death complex.

We then investigated the role of the catalytic activity of caspase‐8 in β‐coronavirus‐induced cell death in human bronchial epithelial cells. Inhibiting the protease activity of caspase‐8 with its specific inhibitor Z‐IETD‐FMK significantly mitigated cell death induced by hCoV‐OC43 infection in Beas‐2B‐ZBP1 cells (Figure 2C,D), highlighting the importance of caspase‐8's catalytic function in β‐coronavirus‐induced cell death. To further delineate the cell death activation in the absence of caspase‐8 catalytic activity, we conducted western blot analysis to assess markers of apoptosis, pyroptosis, and necroptosis. In line with the published results,^[^ ^21^ ^]^ the presence of Z‐IETD‐FMK nearly abolished the activation of caspase‐8 as well as caspase‐3 and ‐7 following hCoV‐OC43 infection (Figure 2E), suggesting the essential role of caspase‐8 in apoptosis induction during β‐coronavirus infection. Moreover, inhibition of caspase‐8 activity markedly reduced the activation of GSDMD (Figure 2F), implying that in coronavirus‐infected bronchial epithelial cells, GSDMD processing is mediated by caspase‐8, which has been discovered in other biological processes.^[^ ^32^, ^33^, ^34^ ^]^ Likewise, cleavage of GSDME, a known substrate of caspase‐3,^[^ ^35^ ^]^ was significantly decreased upon inhibition of caspase‐8 activity (Figure 2F). These results indicate that suppression of caspase‐8 activity can attenuate both apoptosis and pyroptosis induced by β‐coronavirus infection in human bronchial epithelial cells. Additionally, in accordance with the established role of caspase‐8 in inhibiting necroptosis^[^ ^36^, ^37^, ^38^ ^]^ treatment with Z‐IETD‐FMK led to a slight increase in levels of phosphorylated MLKL during hCoV‐OC43 infection (Figure 2G).

This phenotype was further validated in SARS2‐infected human bronchial epithelial cells. Our data indicated that SARS‐CoV‐2 infection was capable of inducing cell death in Beas‐2B‐ZBP1‐EF3‐AT cells, and that inhibition of caspase‐8 activity significantly attenuated the cell death induced by SARS2 infection (Figure 2H,I). Similarly, the suppression of caspase‐8 activation reduced both apoptosis and pyroptosis induced by SARS2 infection, while concurrently exacerbating necroptosis (Figure 2J–L).

Altogether, these results underscore that caspase‐8 is a key component of the ZBP1‐initiated cell death complex in response to β‐coronavirus infection. Its catalytic activity is essential for the induction of both apoptosis and pyroptosis in human bronchial epithelial cells during this process.

### 2.3 NSP5 and ORF6 of SARS‐CoV‐2 Inhibit the Activation of Caspase‐8

Given the essential role of programmed cell death in eliminating infected cells and the critical involvement of caspase‐8 catalytic activity in SARS2‐induced cell death, we next sought to determine whether the virus has evolved mechanisms to inhibit caspase‐8 activation. The genome of SARS2 encodes ≈28 viral proteins, including 16 non‐structural proteins (NSP1‐16), 4 structural proteins (S, E, M, and NP), and 8 accessory proteins (ORF3, ORF6, ORF7a, ORF7b, ORF8, ORF9b, ORF9c, and ORF10).^[^ ^39^ ^]^ To identify potential inhibitors of caspase‐8 encoded by SARS2, we carried out a co‐transfection assay to evaluate the ability of these viral proteins to inhibit the auto‐cleavage of caspase‐8. As expected, the wild‐type caspase‐8 underwent auto‐activation (Figure S3A–D, Supporting Information), consistent with previous reports^[^ ^40^ ^]^ while the catalytic dead mutant (caspase‐8‐C/A) served as a negative control. Our results revealed that several viral proteins, including ORF3, S, E, M, and NP, facilitated the activation of caspase‐8 (Figure S3C,D, Supporting Information), consistent with their documented roles in promoting apoptosis upon overexpression.^[^ ^41^, ^42^, ^43^, ^44^, ^45^, ^46^, ^47^ ^]^ In contrast, NSP1, NSP3C (the C‐terminal domain of NSP3), NSP5, and ORF6 were found to suppress caspase‐8 activation following transfection (Figure S3A,C, Supporting Information). Notably, NSP1 has been shown to shut off host gene expression^[^ ^48^, ^49^ ^]^ as evidenced by the reduced levels of the pro‐form of caspase‐8 observed in NSP1‐transfected cells (Figure S3A, Supporting Information). Similarly, decreased caspase‐8 expression was observed in cells transfected with NSP3C (Figure S3A, Supporting Information), suggesting that the C‐terminal domain of NSP3 also interferes with host gene expression. Based on these findings, we focused our subsequent analyses on the roles of NSP5 and ORF6 in modulating caspase‐8 activation.

### 2.4 3CL‐Pro of Coronaviruses Impede Caspase‐8 Mediated Immune Responses

**Figure 3:**
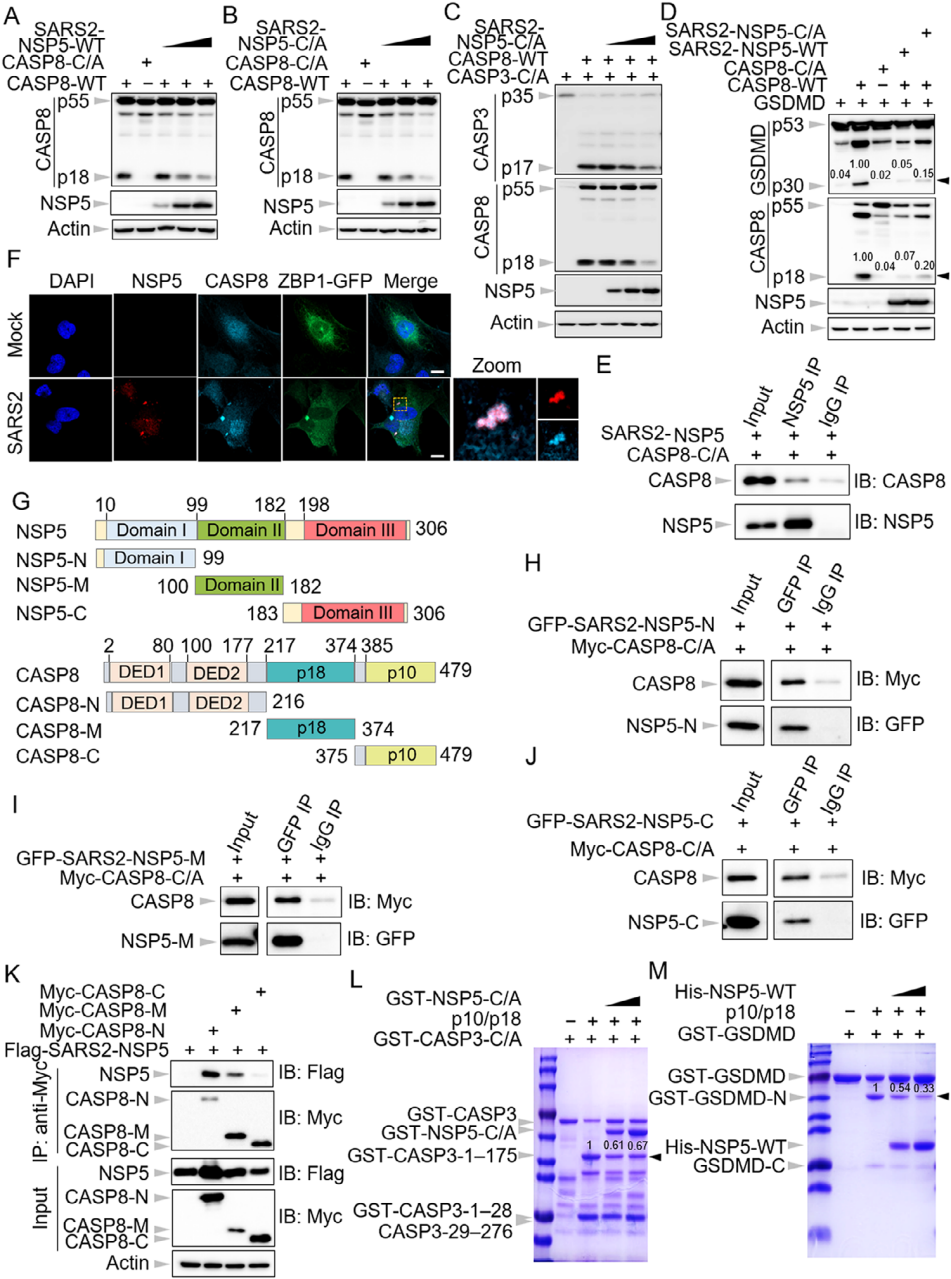
NSP5 of SARS‐CoV‐2 prefers to bind to the large subunit of caspase‐8 to suppress its activation. A,B) Immunoblot analysis of pro‐ and cleaved forms of caspase‐8 (CASP8) after co‐transfection with wild‐type SARS‐CoV‐2 NSP5 (SARS2‐NSP5‐WT) (A) or catalytic dead mutant of SARS2‐NSP5 (SARS2‐NSP5‐C/A) (B) in HEK293T cells. Actin is used as an internal control. C) Immunoblot analysis of pro‐ and cleaved forms of caspase‐3 (CASP3) and CASP8 after co‐transfection with SARS2‐NSP5‐C/A in HEK293T cells. Actin is used as an internal control. D) Immunoblot analysis of pro‐ and cleaved forms of gasdermin D (GSDMD) and CASP8 after co‐transfection with SARS2‐NSP5‐C/A in HEK293T cells. Actin is used as an internal control. E) Immunoprecipitates and total inputs after co‐incubation of purified SARS2 NSP5 and CASP8‐C/A for 16 h. F) Confocal images of CASP8 knock‐out Beas‐2B‐ZBP1‐E‐ORF3‐ACE2‐TMPRSS2 cells overexpressing Myc‐CASP8 (Beas‐2B‐ZBP1‐EF3‐AT + Myc‐CASP8) after infection with SARS2 for 20 h. Scale bar, 10 µm. G) Schematic depiction of the domains in SARS2 NSP5 and CASP8 in the full‐length construct, N‐terminal construct (N), mid‐construct (M), and C‐terminal construct (C). H–J) Immunoprecipitates and total lysates from HEK293T cells after co‐transfection Myc‐CASP8‐C/A with GFP‐SARS2‐NSP5‐N (H), GFP‐SARS2‐NSP5‐M (I), or GFP‐SARS2‐NSP5‐C (J) for 48 h. K) Immunoprecipitates and total lysates from HEK293T cells after co‐transfection Flag‐SARS2‐NSP5 with Myc‐CASP8‐N, Myc‐CASP8‐M, or Myc‐CASP8‐C for 48 h. L,M) In vitro cleavage of purified GST‐tagged caspase‐3 (GST‐CASP3) (L) and gasdermin D (GST‐GSDMD) (M) by active CASP8 p10/p18 tetramer in the presence of increasing doses of SARS2 NSP5. Data are representative of three independent experiments.

To characterize the impact of SARS‐CoV‐2 NSP5 (SARS2‐NSP5) on caspase‐8 activation, we co‐transfected caspase‐8 with increasing doses of SARS2‐NSP5. Our results revealed a dose‐dependent inhibition of caspase‐8 cleavage by SARS2‐NSP5 (**Figure** **3**A). Notably, SARS2‐NSP5, also known as the 3‐chymotrypsin‐like protease (3CL‐pro), is essential to process the viral polyprotein of SARS2, a function conserved across coronaviruses.^[^ ^50^ ^]^ To further investigate whether 3CL‐pros from other coronaviruses could similarly impede caspase‐8 activation, we cloned the 3CL‐pro from several common human and mouse coronaviruses, including hCoV‐OC43, hCoV‐229E, hCoV‐NL63, and mouse hepatitis virus (MHV). Our results demonstrated that all tested 3CL‐pros effectively inhibited the auto‐cleavage of caspase‐8 (Figure S4A–D, Supporting Information), suggesting that this inhibitory effect on caspase‐8 activation is a conserved feature across coronaviruses.

We next investigated the requirement of 3CL‐pro's protease activity for suppressing caspase‐8 activation. The protease function of SARS2‐NSP5 was assessed using a mutant form of gasdermin A (GSDMA) (Figure S4E,F, Supporting Information), in which a consensus cleavage site for coronavirus 3CL‐pro was inserted between the N‐ and C‐terminal domains of GSDMA (GSDMA‐3CL‐pro‐CS).^[^ ^51^ ^]^ Mutagenesis of the catalytic cysteine residue to alanine (C145A) in SARS2‐NSP5 (SARS2‐NSP5‐C/A) resulted in the loss of protease activity (Figure S4F, Supporting Information), in line with previous reports.^[^ ^52^, ^53^ ^]^ Despite the loss of protease activity, co‐transfection with caspase‐8 revealed that SARS2‐NSP5‐C/A still inhibited caspase‐8 activation (Figure 3B). Similarly, a catalytically inactive mutant of hCoV‐OC43 3CL‐pro (hCoV‐OC43‐3CL‐pro‐C/A) was generated, and this mutant also retained its ability to suppress caspase‐8 activation (Figure S4G, Supporting Information). These findings suggest that the protease activity of the coronavirus 3CL‐pro is dispensable for suppressing caspase‐8 activation.

Our investigation into the role of caspase‐8 in mediating apoptosis and pyroptosis during β‐coronavirus infection led us to explore whether SARS2‐NSP5 could inhibit the activation of these pathways. We co‐transfected caspase‐3 and GSDMD with caspase‐8 and SARS2‐NSP5, respectively. A catalytic‐dead mutant of caspase‐3 was employed to prevent its auto‐activation. The results revealed a dose‐dependent inhibition of caspase‐3 cleavage by caspase‐8 in the presence of SARS2‐NSP5 (Figure S4H, Supporting Information). Consistently, this process did not require the catalytic activity of SARS2‐NSP5 (Figure 3C). To further investigate whether the 3CL‐pro of coronaviruses could suppress apoptosis activation during viral infection, we evaluated apoptosis induction during IAV infection in the presence of SARS2‐NSP5 and hCoV‐OC43‐3CL‐pro, respectively. Notably, IAV infection can also trigger ZBP1‐mediated cell death through the recruitment of RIPK3 and caspase‐8.^[^ ^15^, ^16^, ^54^ ^]^ To facilitate the detection of cell death following IAV infection in HEK293 cells, we developed a 293‐RIPK3‐ZBP1 cell line with doxycycline (Dox)‐inducible expression of ZBP1 (Figure S4I, Supporting Information). Real‐time cell death analysis confirmed that ZBP1 augmentation in this cell line exacerbated cell death following IAV infection (Figure S4J, Supporting Information). Moreover, cleavage of caspase‐8, ‐7, and ‐3 was observed, indicating the induction of apoptosis following infection (Figure S4K, Supporting Information). Notably, both SARS2‐NSP5 and hCoV‐OC43‐3CL‐pro were found to attenuate the activation of apoptosis triggered by IAV infection (Figure S4K, Supporting Information). Furthermore, SARS2‐NSP5 was found to inhibit caspase‐8‐mediated GSDMD activation independently of its protease activity (Figure 3D).

Collectively, the results above indicate that the 3CL‐pros of coronaviruses function as a potent inhibitor of caspase‐8, thereby effectively dampening caspase‐8‐mediated immune responses.

### 2.5 NSP5 of SARS‐CoV‐2 Prefers to Bind to the Large Subunit of Caspase‐8 to Inhibit its Activity

To investigate the mechanism by which SARS2‐NSP5 inhibits caspase‐8 activation, we first assessed the interaction between SARS2‐NSP5 and caspase‐8 via the co‐immunoprecipitation assay. To minimize potential cell death due to overexpression, we employed the catalytic‐dead mutant of caspase‐8. Our results revealed that SARS2‐NSP5 specifically interacts with caspase‐8, as evidenced by the co‐immunoprecipitation of caspase‐8‐C/A, instead of caspase‐1, with SARS2‐NSP5 (Figure S5A, Supporting Information), and vice versa (Figure S5B, Supporting Information). To determine whether this interaction is direct, we tested the binding between purified proteins. Notably, purified caspase‐8‐C/A was successfully immunoprecipitated by purified SARS2‐NSP5 after co‐incubation (Figure 3E), confirming a direct interaction between SARS2‐NSP5 and caspase‐8. Additionally, we observed that 3CL‐pro from both hCoV‐OC43 and MHV also interacts with caspase‐8 (Figure S5C,D, Supporting Information), implying a conserved function among β‐coronavirus 3CL‐pros in interacting with caspase‐8. The interaction between SARS2‐NSP5 and caspase‐8 was further validated by immunofluorescence microscopy following SARS2 infection. We observed puncta of SARS2‐NSP5 formed in infected cells and co‐localized with caspase‐8 (Figure 3F).

SARS2‐NSP5 is composed of three domains: domain I, domain II, and domain III,^[^ ^55^ ^]^ while caspase‐8 consists of two tandem death effector domains (DEDs) and a catalytic domain comprising a large subunit (p18) and a small subunit (p10)^[^ ^56^ ^]^ (Figure 3G). To map the domains required for the interaction between SARS2‐NSP5 and caspase‐8, we generated truncation mutants of both proteins (Figure 3G). Remarkably, all three domains of SARS2‐NSP5 were capable of binding to full‐length caspase‐8 (Figure 3H–J). Similarly, each truncation mutant of caspase‐8 was able to interact with SARS2‐NSP5, with the large subunit of caspase‐8 showing a significantly stronger binding capacity for SARS2‐NSP5 compared to its small subunit (Figure 3K). To determine whether this interaction is sufficient to inhibit the auto‐cleavage of caspase‐8, we co‐transfected the truncation mutants with caspase‐8. Notably, while full‐length SARS2‐NSP5 consistently demonstrated robust inhibition of caspase‐8 cleavage, all three domains of SARS2‐NSP5 exhibited comparable inhibitory effects on caspase‐8 activation (Figure S5E, Supporting Information), partially explaining why the protease activity of SARS2‐NSP5 is dispensable for the suppression of caspase‐8 activation (Figure 3B–D).

Given the direct binding capacity of SARS2‐NSP5 to caspase‐8 and the apparent sufficiency of this interaction to inhibit caspase‐8 activation, we next sought to examine whether SARS2‐NSP5 could also affect the catalytic activity of caspase‐8 in vitro. To address this, we performed an in vitro cleavage assay using active caspase‐8 in the presence of purified SARS2‐NSP5‐C/A. Purified caspase‐3‐C/A and GSDMD were employed as substrates for caspase‐8, with the cleavage sites for both substrates annotated (Figure S5F, Supporting Information). Upon incubation with the caspase‐8 p10/p18 tetramer, both caspase‐3‐C/A and GSDMD were cleaved, leading to a reduction of their pro‐forms and an increase in the cleaved fragments (Figure S5G,H, Supporting Information). We then added increasing amounts of SARS2‐NSP5‐C/A to the reaction and observed a significant inhibition of caspase‐3‐C/A cleavage by active caspase‐8 (Figure 3L). In contrast, the addition of GST, as a negative control, had little effect on caspase‐3‐C/A cleavage (Figure S5G, Supporting Information), suggesting that the observed inhibition of caspase‐8 activity is specifically mediated by SARS2‐NSP5‐C/A. Due to similar molecular weights of GST‐NSP5‐C/A and GST‐GSDMD‐N, we employed His‐NSP5‐WT for the GSDMD cleavage assay instead. Similarly, SARS2‐NSP5‐WT inhibited the cleavage of GSDMD by caspase‐8 in a dose‐dependent manner (Figure 3M), while GST had no effect (Figure S5H, Supporting Information). Taken together, the data here indicate that SARS2‐NSP5 can directly bind to caspase‐8, most likely targeting its large subunit, thereby inhibiting its catalytic activity.

### 2.6 The ORF6 of SARS‐CoV‐2 Restrains Caspase‐8‐Mediated Immune Responses

In a similar vein, we examined the potential of SARS‐CoV‐2 ORF6 (SARS2‐ORF6) to inhibit caspase‐8 activation. Our results demonstrated that SARS2‐ORF6 effectively suppressed caspase‐8 auto‐cleavage in a dose‐dependent manner (Figure S6A, Supporting Information). Furthermore, SARS2‐ORF6 attenuated caspase‐8‐mediated apoptosis, as evidenced by the diminished cleavage of caspase‐3 in the presence of SARS2‐ORF6 (Figure S6B, Supporting Information). Additionally, SARS2‐ORF6 was shown to inhibit apoptosis induced by IAV infection (Figure S6C, Supporting Information). Moreover, SARS2‐ORF6 was also found to obstruct caspase‐8‐mediated cleavage of GSDMD (Figure S6D, Supporting Information), suggesting its role in inhibiting caspase‐8‐driven pyroptosis. In sum, these findings highlight the capacity of SARS2‐ORF6 to impede caspase‐8‐mediated immune responses.

### 2.7 The ORF6 C‐Terminus of SARS‐CoV‐2 is Required to Interact with Caspase‐8

**Figure 4:**
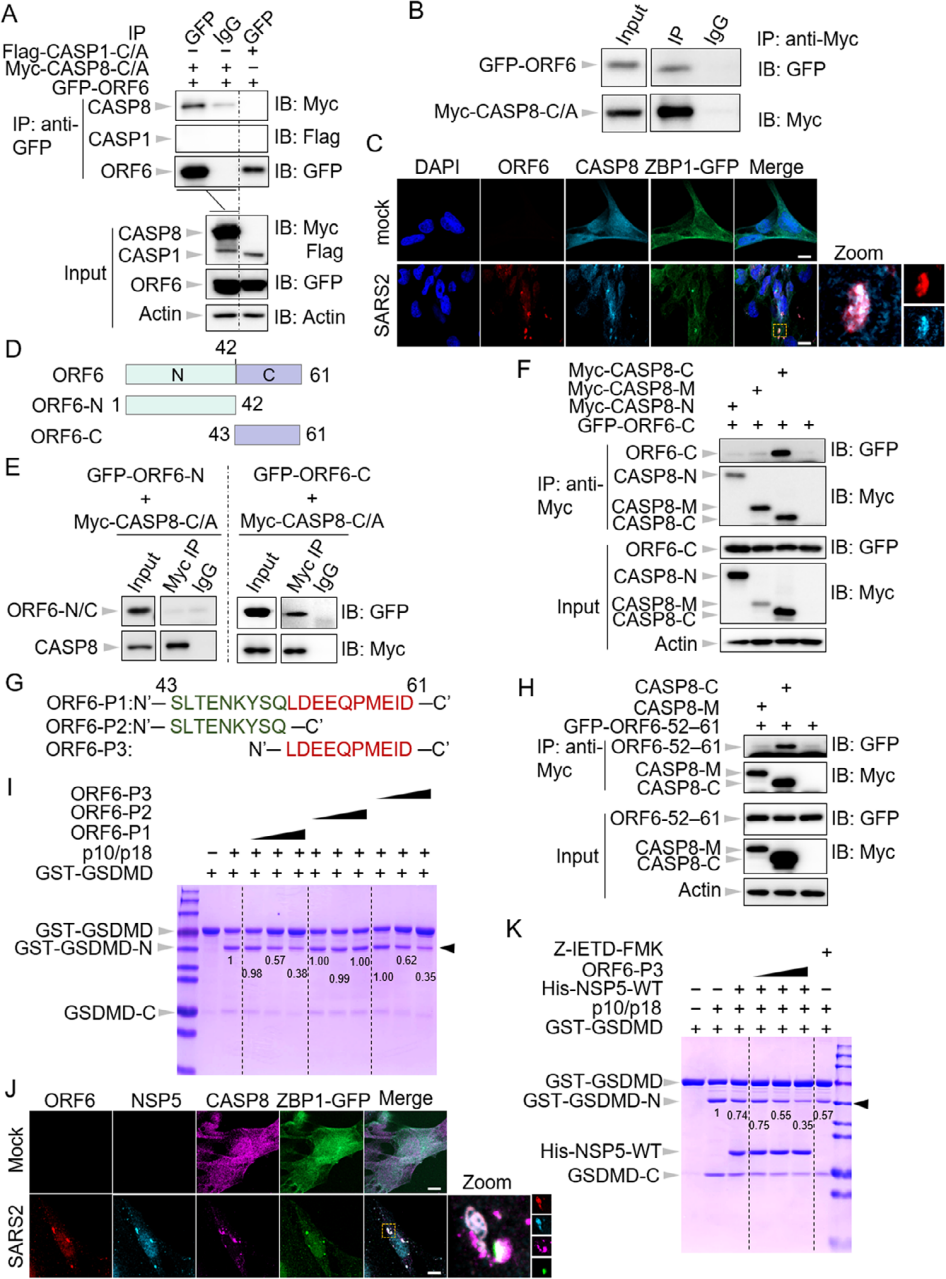
The ORF6 C‐terminus of SARS‐CoV‐2 binds to the small subunit of caspase‐8 to inhibit its activity. A) Immunoprecipitates and total lysates from HEK293T cells after co‐transfection of GFP‐ORF6, Flag‐caspase‐1‐C/A (Flag‐CASP1‐C/A), or Myc‐caspase‐8‐C/A (Myc‐CASP8‐C/A) for 48 h. B) Immunoprecipitates and total lysates from HEK293T cells after co‐transfection of GFP‐ORF6 and Myc‐CASP8‐C/A for 48 h. C) Confocal images of CASP8 knock‐out Beas‐2B‐ZBP1‐E‐ORF3‐ACE2‐TMPRSS2 cells overexpressing Myc‐CASP8 (Beas‐2B‐ZBP1‐EF3‐AT + Myc‐CASP8) after infection with SARS‐CoV‐2 (SARS2) for 20 h. Scale bar, 10 µm. D) Schematic depiction of the domains in SARS2 ORF6 in the full‐length construct, N‐terminal construct (N), and C‐terminal construct (C). E) Immunoprecipitates and total lysates from HEK293T cells after co‐transfection of GFP‐ORF6‐N or GFP‐ORF6‐C with Myc‐CASP8‐C/A for 48 h. F) Immunoprecipitates and total lysates from HEK293T cells after co‐transfection GFP‐ORF6‐C with Myc‐CASP8‐N, Myc‐CASP8‐M, or Myc‐CASP8‐C for 48 h. G) Peptide sequences of SARS2 ORF6‐C (ORF6‐P1), ORF6‐43–51 (ORF6‐P2), and ORF6‐52–61 (ORF6‐P3). H) Immunoprecipitates and total lysates from HEK293T cells after co‐transfection GFP‐ORF6‐52–61 with Myc‐CASP8‐M or Myc‐CASP8‐C for 48 h. I) In vitro cleavage of purified GST‐tagged gasdermin D (GST‐GSDMD) by active CASP8 p10/p18 tetramer in the presence of increasing doses of SARS2 ORF6‐P1, ORF6‐P2, or ORF6‐P3. J) Confocal images of CASP8 knock‐out Beas‐2B‐ZBP1‐E‐ORF3‐ACE2‐TMPRSS2 cells overexpressing Myc‐CASP8 (Beas‐2B‐ZBP1‐EF3‐AT + Myc‐CASP8) after infection with SARS2 encoding Flag‐tagged ORF6 for 20 h. Scale bar, 10 µm. K) In vitro cleavage of purified GST‐GSDMD by active CASP8 p10/p18 tetramer in the presence of SARS2 NSP5 (1 µg) and increasing doses of ORF6‐P3 (1, 2, and 4 µg) or Z‐IETD‐FMK (5 mm). Data are representative of three independent experiments.

To elucidate the mechanism by which SARS2‐ORF6 inhibits caspase‐8 activation, we conducted a co‐immunoprecipitation assay following the co‐transfection of ORF6 with caspase‐8‐C/A. Our results demonstrated that ORF6 specifically interacted with caspase‐8, as indicated by the pull‐down of caspase‐8 by ORF6, and vice versa, with no interaction observed between ORF6 and caspase‐1 (**Figure** **4**A,B). Consistent with these findings, we observed co‐localization of SARS2‐ORF6 and caspase‐8 following SARS2 infection (Figure 4C). To further investigate the interaction, we generated truncation mutants to map the domains responsible for this binding. SARS2‐ORF6 is composed of an N‐terminal domain and a C‐terminal domain^[^ ^57^ ^]^ (Figure 4D). Upon co‐transfection with caspase‐8‐C/A, only the C‐terminal domain of SARS2‐ORF6 was immunoprecipitated by caspase‐8‐C/A (Figure 4E), suggesting that the C‐terminal domain is critical for its interaction with caspase‐8. To further assess this interaction, we performed a GST‐pull down assay using purified proteins to determine whether the C‐terminal domain of SARS2‐ORF6 directly binds to caspase‐8. In line with the findings from the overexpression system, the purified N‐terminal domain of SARS2‐ORF6 did not interact with caspase‐8, whereas the C‐terminal domain effectively pulled down caspase‐8 (Figure S7A, Supporting Information). Additionally, the C‐terminal domain of SARS2‐ORF6 inhibited caspase‐8 activation, whereas the N‐terminal domain had no effect on caspase‐8 auto‐cleavage (Figure S7B, Supporting Information). These findings underscore the importance of the C‐terminal domain of SARS2‐ORF6 in modulating caspase‐8 activity.

We next identified the specific domain of caspase‐8 required for its interaction with SARS2‐ORF6, revealing that the small subunit of caspase‐8 mediates this interaction (Figure 4F), in stark contrast to the interaction observed with SARS2‐NSP5. To further elucidate the peptide regions of SARS2‐ORF6 involved in binding to caspase‐8, we divided the C‐terminal domain (ORF6‐P1) of SARS2‐ORF6 into two segments based on its crystal structure:^[^ ^58^ ^]^ ORF6‐43–51 (ORF6‐P2), comprising residues 43–51, and ORF6‐52–61 (ORF6‐P3), encompassing residues 52–61 (Figure 4G). Upon co‐transfection with caspase‐8, we found that only ORF6‐P3 effectively inhibited caspase‐8 activation (Figure S7C, Supporting Information), suggesting that ORF6‐P3 mediates the interaction with caspase‐8. In line with this, co‐immunoprecipitation analysis showed that ORF6‐P3 specifically bound to the small subunit of caspase‐8, rather than the large subunit (Figure 4H), further supporting the essential role of the small subunit in the interaction with SARS2‐ORF6.

Given that the C‐terminal domain of SARS2‐ORF6 can directly interact with caspase‐8 (Figure S7A, Supporting Information), it is plausible that it inhibits the catalytic activity of caspase‐8 in vitro, akin to the effect observed with SARS2‐NSP5. To test this hypothesis, we synthesized three peptides from SARS2‐ORF6: ORF6‐P1, ORF6‐P2, and ORF6‐P3 (Figure 4G). Consistent with the overexpression data, both ORF6‐P1 and ‐P3 were able to obstruct the caspase‐8 p10/p18 tetramer‐mediated cleavage of caspase‐3 and GSDMD, whereas ORF6‐P2 did not affect the cleavage of these substrates by caspase‐8 (Figure 4I,D).

Given that the distinct binding preference of SARS2‐NSP5 and ORF6 for different subunits of caspase‐8, we hypothesized that these proteins might cooperate to inhibit caspase‐8 activation during infection. To investigate this, we first assessed the co‐localization of NSP5 and ORF6 following SARS2 infection. Remarkably, we observed robust co‐localization of SARS2‐NSP5 and ‐ORF6 in infected cells (Figure S7E, Supporting Information), along with the formation of complexes between both proteins and caspase‐8 (Figure 4J), suggesting a potential synergistic inhibition of caspase‐8 activity by NSP5 and ORF6. To further test this hypothesis, we performed an in vitro cleavage assay in the presence of both SARS2‐NSP5 and ORF6‐P3. As expected, the combination of NSP5 and ORF6 enhanced the inhibition of caspase‐8 catalytic activity to a degree comparable to the effect of the caspase‐8 inhibitor Z‐IETD‐FMK (Figure 4K). This finding implies a synergistic action of SARS2‐NSP5 and ‐ORF6 in suppressing caspase‐8‐mediated immune responses.

To sum up, all the data here demonstrate that the C‐terminal domain of SARS2‐ORF6 directly interacts with the small subunit of caspase‐8 to inhibit its catalytic activity, and that SARS2‐ORF6 and ‐NSP5 act cooperatively to hinder caspase‐8 function.

### 2.8 NSP13 of Coronavirus Can Inhibit ZBP1‐Induced Necroptosis

**Figure 5:**
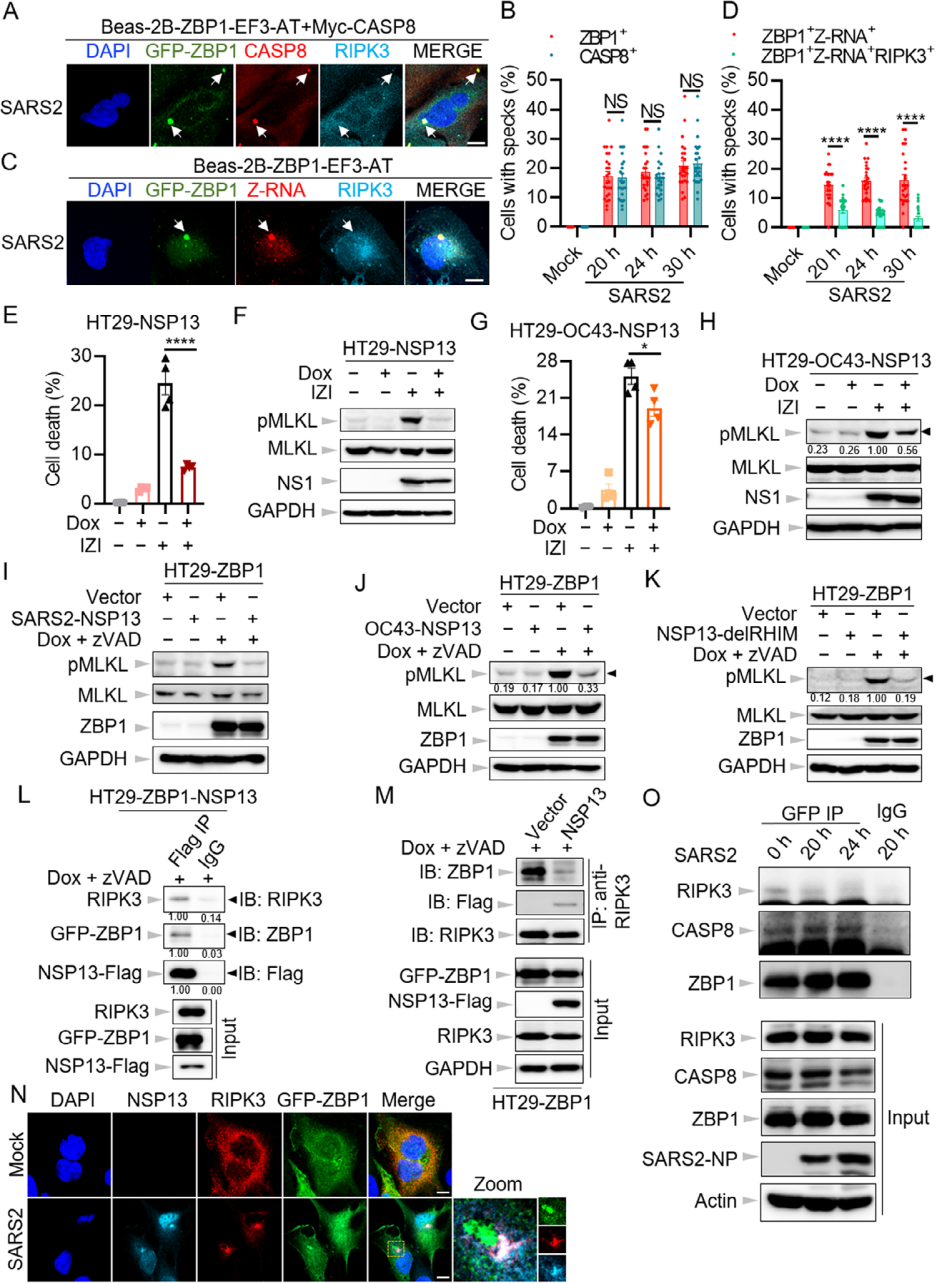
NSP13 of coronavirus hijacks RIPK3 to impede ZBP1‐initiated necroptosis. A) Confocal images of caspase‐8 (CASP8) knock‐out Beas‐2B‐ZBP1‐E‐ORF3‐ACE2‐TMPRSS2 cells overexpressing Myc‐CASP8 (Beas‐2B‐ZBP1‐EF3‐AT + Myc‐CASP8) after infection with SARS‐CoV‐2 (SARS2). Scale bar, 10 µm. B) Quantification of the percentage of cells with ZBP1^+^ or CASP8^+^ specks in A for the indicated time points. C) Confocal images of Beas‐2B‐ZBP1‐EF3‐AT cells infected with SARS2. Scale bar, 10 µm. D) Quantification of the percentage of cells with ZBP1^+^Z‐RNA^+^ or ZBP1^+^Z‐RNA^+^RIPK3^+^ specks in C for the indicated time points. E) Cell death analysis by IncuCyte in HT29‐NSP13 cells infected by influenza A virus (IAV) together with zVAD plus interferon‐α2b (IFN‐α2b) (IZI) in the presence of 500 ng mL^−1^ doxycycline (Dox) for 20 h. F) Immunoblot analysis of phosphorylated mixed lineage kinase domain‐like protein (pMLKL), total MLKL, and influenza NS1 protein in (E). GAPDH is used as the internal control. G) Cell death analysis by IncuCyte in HT29‐OC43‐NSP13 cells treated with IZI in the presence of 500 ng mL^−1^ Dox for 20 h. H) Immunoblot analysis of pMLKL, total MLKL, and influenza NS1 protein in (G). GAPDH is used as the internal control. I–K) Immunoblot analysis of pMLKL, total MLKL, and ZBP1 in HT29‐ZBP1‐NSP13 (I), HT29‐ZBP1‐OC43‐NSP13 (J), and HT29‐ZBP1‐NSP13‐delRHIM (K) cells in the presence of 500 ng mL^−1^ Dox and zVAD for 20 h. GAPDH is used as the internal control. L,M) Immunoprecipitates and total lysates from HT29‐ZBP1‐NSP13 cells with Flag IP (L) or RIPK3 IP (M) in the presence of 500 ng mL^−1^ Dox and zVAD for 20 h. O) Immunoprecipitates and total lysates from Beas‐2B‐ZBP1‐EF3‐AT cells overexpressing HA‐RIPK3 after infection with SARS2 for the indicated time points. N) Confocal images of Beas‐2B‐ZBP1‐EF3‐AT cells infected with SARS2. Scale bar, 10 µm. NS, not significant; *P < 0.05; ****P < 0.0001. Analysis was performed using two‐way ANOVA (B,D) or one‐way ANOVA (E,G). Data are shown as mean ± SEM (*n* = 25) (B,D) or (*n* = 4) (E,G). Data are representative of three independent experiments.

Necroptosis has long been considered as a ‘trap door’ alternative to extrinsic apoptosis during viral infections, as blocking the activity of caspase‐8 can promote necroptosis.^[^ ^59^, ^60^ ^]^ Consequently, the inhibition of caspase‐8 activation by SARS2‐NSP5 and ‐ORF6 could potentially redirect the virus toward this pro‐necrotic pathway. However, our confocal microscopy analysis revealed an intriguing trend in cells infected with hCoV‐OC43 and SARS2: while ZBP1‐positive specks consistently contained caspase‐8, the presence of RIPK3 within these specks progressively diminished over time (**Figure** **5**A–D; Figure S8A–D, Supporting Information). This observation suggests that β‐coronavirus may inhibit the recruitment of RIPK3 to ZBP1‐initiated cell death complexes. Based on this, we hypothesized that β‐coronavirus may interfere with the activation of necroptosis by targeting RIPK3, the essential kinase required for MLKL activation.^[^ ^61^ ^]^ To explore this further, we co‐transfected all SARS2 non‐structural and accessory proteins with RIPK3 for co‐localization analysis via immunofluorescence microscopy, with the exception of NSP3 due to the unavailability of a full‐length clone. Our results provided compelling evidence of colocalization between several viral proteins, including SARS2‐NSP6, ‐NSP13, and ‐NSP14, with RIPK3 (Figure S8E, Supporting Information).

To verify whether these proteins can inhibit necroptosis activation, we established three inducible stable HT29 cell lines, each expressing SARS2‐NSP6, ‐NSP13, or ‐NSP14 upon Dox induction, respectively (Figure S9A–C, Supporting Information). Considering that SARS2‐induced necroptosis is dependent on ZBP1,^[^ ^6^, ^7^ ^]^ we sought to infect these HT29 cell lines with IAV plus zVAD, a combination known to bias IAV‐induced ZBP1‐mediated cell death toward necroptosis.^[^ ^17^, ^18^ ^]^ The expression of endogenous ZBP1 was first confirmed in HT29 cells following stimulation with interferon α2b (IFN‐α2b), using real‐time PCR and western blot analysis (Figure S9D,E, Supporting Information). Subsequently, cells were exposed to IAV along with zVAD and IFN‐α2b (IZI) to assess the activation of necroptosis. Notably, neither SARS2‐NSP6 nor ‐NSP14 was able to prevent IAV‐induced necroptosis under these conditions (Figure S9F–I, Supporting Information). In parallel, phosphorylation of MLKL remained unaffected upon induction of SARS2‐NSP6 or ‐NSP14 following IZI treatment (Figure S9J,K, Supporting Information). In contrast, SARS2‐NSP13 significantly inhibited both IZI‐induced cell death and MLKL phosphorylation (Figure 5E,F; Figure S9L,Supporting Information), implicating that SARS2‐NSP13 can suppress ZBP1‐driven necroptosis. To determine whether this inhibitory effect is conserved across other β‐coronaviruses, we constructed a similar inducible cell line expressing NSP13 from hCoV‐OC43 (OC43‐NSP13) (Figure S9M, Supporting Information). As observed with SARS2‐NSP13, OC43‐NSP13 also inhibited IZI‐induced ZBP1‐mediated necroptosis, although the effect was less pronounced compared to SARS2‐NSP13 (Figure 5G,H; Figure S9N, Supporting Information).

To further confirm the inhibitory effect of NSP13, we introduced NSP13 into an HT29‐ZBP1 cell line, in which both NSP13 and ZBP1 expression were induced by Dox (Figure 5I,J; Figure S9O,P, Supporting Information). Previous studies have demonstrated that Dox plus zVAD can trigger ZBP1‐initiated necroptosis in the HT29‐ZBP1 inducible cell line.^[^ ^62^ ^]^ In accordance with this, we observed MLKL phosphorylation under these conditions in control cells transduced with ZBP1 and an empty vector (Figure 5I). In agreement with our results in IZI‐treated HT29 cells, simultaneous expression of SARS2‐NSP13 significantly inhibited ZBP1‐induced necroptosis (Figure 5I), as did OC43‐NSP13 (Figure 5J). Notably, the expression of ZBP1 remained unchanged upon induction of SARS2‐NSP13 or OC43‐NSP13 (Figure 5I,J), indicating that the observed inhibitory effect was not due to a decrease in ZBP1 expression. These findings provide further evidence that NSP13 from β‐coronaviruses can suppress ZBP1‐induced necroptosis, and this function appears to be conserved across different β‐coronaviruses.

Unexpectedly, while phosphorylated MLKL levels were reduced in the presence of SARS2‐NSP13 or OC43‐NSP13 (Figure 5I,J), cell death was not significantly altered compared to control cells (Figure S9Q,R, Supporting Information). To investigate the nature of cell death activated under these conditions, we treated the cells with dabrafenib (Dabra), a selective RIPK3 kinase inhibitor.^[^ ^63^ ^]^ As anticipated, Dabra completely blocked cell death in control cells (Figure S9Q,R, Supporting Information), confirming that only necroptosis was induced in these cells. However, Dabra only modestly reduced cell death in HT29‐ZBP1 cells expressing SARS2‐NSP13 or OC43‐NSP13 (Figure S9Q,R, Supporting Information), suggesting the activation of an alternative, non‐apoptotic and non‐necroptotic cell death pathway when NSP13 from β‐coronaviruses and ZBP1 are co‐expressed. Altogether, the results above indicate that NSP13 from β‐coronaviruses can effectively inhibit ZBP1‐initiated necroptosis.

### 2.9 NSP13 of Coronavirus Hijacks RIPK3 to Impede ZBP1‐Initiated Necroptosis

A recent study identified a putative RHIM within the 1B domain of SARS2‐NSP13^[^ ^20^ ^]^ (Figure S10A, Supporting Information). This motif, which is shared by RIPK1, RIPK3, and ZBP1, can mediate the interactions between RHIM‐containing proteins. To investigate whether the putative RHIM domain of SARS2‐NSP13 contributes to the inhibition of ZBP1‐initiated necroptosis, we transduced SARS2‐NSP13‐delRHIM into the HT29‐ZBP1 inducible cell line (Figure S10B, Supporting Information). This mutation, lacking the four core amino acids of the RHIM, has been shown to impair interactions between RHIM‐containing proteins.^[^ ^64^, ^65^ ^]^ Our results revealed that MLKL phosphorylation was diminished in cells expressing SARS2‐NSP13‐delRHIM, similar to those expressing the intact SARS2‐NSP13 (Figure 5K). Additionally, although the RHIM of OC43‐NSP13 has been suggested to be non‐functional,^[^ ^20^ ^]^ it still effectively suppressed ZBP1‐induced necroptosis (Figure 5H,J). These findings suggest that the putative RHIM of NSP13 may not be required for inhibiting ZBP1‐induced necroptosis.

To elucidate the underlying molecular mechanisms, we carried out co‐immunoprecipitation assays. Initially, we examined the interaction between SARS2‐NSP13 and RIPK3 in an overexpression system. Consistent with the confocal microscopy findings above (Figure S8E, Supporting Information), we observed that SARS2‐NSP13 interacted with RIPK3, so did OC43‐NSP13 (Figure S10C,D, Supporting Information). Notably, the RHIM‐deleted variant of SARS2‐NSP13 retained its ability to bind to RIPK3 (Figure S10E, Supporting Information). Furthermore, SARS2‐NSP13 successfully immunoprecipitated RIPK3 in the HT29‐ZBP1 inducible cell line after Dox plus zVAD treatment, with ZBP1 also being pulled down in this assay (Figure 5L). To explore the possible explanation for reduced necroptosis in Dox plus zVAD‐treated HT29‐ZBP1 cells when both ZBP1 and NSP13 were induced simultaneously, we checked the interaction between RIPK3 and ZBP1 under these conditions. It was found that the presence of SARS2‐NSP13 diminished the interaction between RIPK3 and ZBP1, and vice versa (Figure 5M; Figure S10F, Supporting Information). Similarly, OC43‐NSP13 also disrupted the binding of RIPK3 to ZBP1 (Figure S10G, Supporting Information). Importantly, the RHIM‐deleted variant of SARS2‐NSP13 retained its ability to interfere with the RIPK3‐ZBP1 interaction (Figure S10H, Supporting Information). These results suggest that NSP13 from β‐coronaviruses can disrupt the interaction between RIPK3 and ZBP1, independent of its putative RHIM motif. Consistent with these findings, we also observed that NSP13 formed puncta with RIPK3 that did not co‐localize with ZBP1 following SARS2 infection (Figure 5N). Additionally, co‐immunoprecipitation assay revealed that, in response to SARS2 infection, the interaction between RIPK3 and ZBP1 decreased over time (Figure 5O). Taken together, the data here indicate that β‐coronavirus NSP13 interacts with RIPK3, thereby hindering its recruitment into the ZBP1‐induced cell death complex and suppressing ZBP1‐initiated necroptosis.

### 2.10 All Domains of SARS2‐CoV‐2 NSP13 Suppress ZBP1‐Initiated Necroptosis

To delineate the specific domains of SARS2‐NSP13 that interact with RIPK3, we co‐transfected each individual domain of SARS2‐NSP13 with RIPK3 and performed co‐immunoprecipitation assays. Surprisingly, we found that all domains of SARS2‐NSP13 were capable of interacting with RIPK3 (Figure S11A–E, Supporting Information). To verify whether these interactions were sufficient to inhibit ZBP1‐initiated necroptosis, we generated stable HT29‐ZBP1 cell lines with Dox‐inducible expression of each SARS2‐NSP13 domain (Figure S11F–J, Supporting Information). Upon induction, all NSP13 domains effectively inhibited ZBP1‐initiated necroptosis, as evidenced by reduced phosphorylation of MLKL (Figure S11K–O, Supporting Information). These results further confirm that the inhibitory effect of SARS2‐NSP13 on ZBP1‐mediated necroptosis is independent of its putative RHIM domain. In summary, these findings strongly suggest that all domains of SARS2‐NSP13 function as potent inhibitors of ZBP1‐initiated necroptosis.

### 2.11 Co‐Infection of Influenza A Virus and Coronavirus Induces Robust Inflammatory Responses Due to Partial Cell Death Inhibition by Coronavirus

Clinical studies have demonstrated that co‐infection with influenza viruses and SARS2 can exacerbate disease severity and increase mortality rates.^[^ ^66^, ^67^ ^]^ Studies in mice have shown that co‐infection with IAV and SARS2 results in enhanced IAV replication and more pronounced inflammatory responses in the lungs.^[^ ^68^ ^]^ However, the mechanisms underlying this remain poorly understood.

To investigate this phenomenon further, we co‐infected Beas‐2B‐ZBP1 cells with IAV and OC43 and observed increased replication of both viruses in this human bronchial epithelial cell line (Figure S12A,B, Supporting Information). Additionally, co‐infected cells exhibited elevated expression of inflammatory cytokines, including *IL‐6*, *TNF*, and *IL1β* (Figure S12C–E, Supporting Information), alongside increased activation of the type I IFN pathway (Figure S12F,G, Supporting Information).

**Figure 6:**
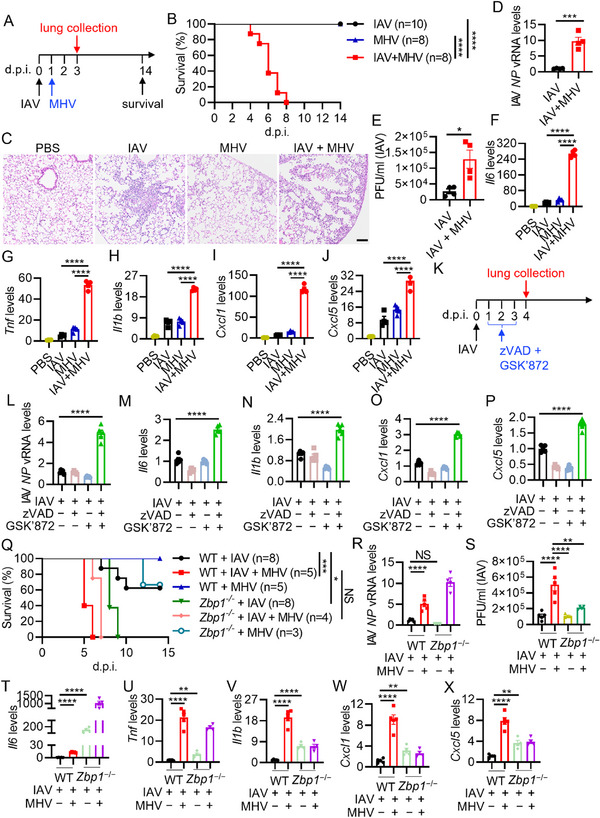
Co‐infection of influenza A virus and coronavirus induces robust inflammatory responses due to partial cell death inhibition by coronavirus. A) Schematic diagram of the experiment design. Six to eight‐week‐old mice challenged with 100 plaque‐forming units (PFU) of influenza A virus (IAV) A/PR8/34 (PR8) were infected with mouse hepatitis virus (MHV) at day one post‐IAV infection. Lung samples were collected at day three post‐IAV infection or day two post‐MHV infection. Body weight was monitored daily for two weeks. B) Survival of mice infected with the indicated viruses. C) Representative images from the lung sections of the indicated mice stained with H&E (scale bar, 100 µm). D) Real‐time PCR analysis of the expression of IAV NP vRNA levels in the lungs of mice infected with the indicated viruses, presented relative to levels of the host gene *18s rRNA*. E) Lung IAV titers in mice infected with the indicated viruses. F–J) Real‐time PCR analysis of the expression of *Il6* (F), *Tnf* (G), *Il1b* (H), *Cxcl1* (I), and *Cxcl5* (J) in the lungs of mice with the indicated treatment, presented relative to levels of the host gene *18s rRNA*. K) Schematic diagram of the experiment design. Six to eight‐week‐old mice challenged with 100 PFU of IAV PR8 were subjected to the administration of zVAD, GSK’872, or zVAD + GSK’872 via intraperitoneal injection once daily for three days. Lung samples were collected at day four post‐IAV infection. Body weight was monitored daily for two weeks. L–P) Real‐time PCR analysis of the expression of IAV NP vRNA levels (L), *Il6* (M), *Il1b* (N), *Cxcl1* (O), and *Cxcl5* (P) in the lungs of mice with the indicated treatment, presented relative to levels of the host gene *18s rRNA*. Q) Survival of mice infected with the indicated viruses, and in this panel, 120 PFU of PR8 was inoculated per mouse. R) Real‐time PCR analysis of the expression of IAV NP vRNA levels in the lungs of mice infected with the indicated viruses, presented relative to levels of the host gene *18s rRNA*. S) Lung IAV titers in mice infected with the indicated viruses. T–X) Real‐time PCR analysis of the expression of *Il6* (T), *Tnf* (U), *Il1b* (V), *Cxcl1* (W), and *Cxcl5* (X) in the lungs of indicated mice, presented relative to levels of the host gene *18s rRNA*. NS, not significant; *P < 0.05, **P < 0.01, **P < 0.001, and ****P < 0.0001. Analysis was performed using Student's *t*‐test (D,E), one‐way ANOVA (F–J, and L–P), two‐way ANOVA (R–X), and log‐rank test (B,Q). Data are shown as mean ± SEM (*n* = 4) (D–J), (*n* = 5) (L–P), (*n* = 4–5) (R–X) or (*n* = 3–10) (B,Q). Data are representative of three independent experiments.

To extend these findings to an in vivo setting, we sequentially infected mice with IAV followed by mouse hepatitis virus (MHV), a prototypical β‐coronavirus, to investigate the interaction between the host and coronavirus^[^ ^69^ ^]^ (**Figure** **6**A). Consistently, co‐infection of IAV and MHV significantly increased mortality in infected mice (Figure 6B). The lungs of co‐infected mice showed heightened inflammation (Figure 6C). Moreover, we detected elevated levels of IAV vRNA and viral particles in the lungs of co‐infected mice (Figure 6D,E), indicating enhanced IAV replication, while MHV replication remained unchanged (Figure S12H,I, Supporting Information). Inflammatory cytokines and chemokines, including *Il6*, *Tnf*, *Il1b*, *Cxcl1*, and *Cxcl5*, were also significantly upregulated in the lungs of co‐infected mice (Figure 6F–J). Similarly, elevated type I IFN responses were observed in co‐infected mice (Figure S12J,K, Supporting Information). Collectively, these data further support the published results that co‐infection with IAV and β‐coronavirus promotes IAV replication and triggers a robust inflammatory response in vivo.

Extensive studies have revealed the complex interplay between cell death pathways and innate immune sensing mechanisms.^[^ ^16^, ^70^, ^71^, ^72^, ^73^, ^74^, ^75^, ^76^ ^]^ For instance, the activation of intrinsic apoptosis leads to the release of mitochondrial DNA, which in turn activates the cGAS‐STING pathway. Concurrently, apoptotic caspases can cleave cGAS and MAVS, thereby inhibiting the activation of both cGAS and RIG‐I‐like receptors initiated innate immune responses.^[^ ^70^, ^72^ ^]^ Given the inhibitory effect of coronavirus on cell death activation, we hypothesized that this inhibition might promote IAV replication and exacerbate infection‐induced inflammatory responses. To test this hypothesis, we treated IAV‐infected cells with the pan‐caspase inhibitor zVAD and the RIPK3 kinase‐specific inhibitor GSK’872 to mimic cell death inhibition by coronavirus. Initially, we applied a high concentration of zVAD (20 µm) in combination with GSK’872. Consistent with previous findings highlighting the essential role of apoptotic caspases in exporting the IAV genome from the nucleus,^[^ ^77^, ^78^ ^]^ this combination significantly reduced IAV replication (Figure S12L, Supporting Information). Since coronavirus can still induce cell death in the presence of cell death inhibitors encoded, suggesting only partial inhibition during infection, we lowered the dose of zVAD. Surprisingly, we observed a substantial increase in IAV replication in human bronchial epithelial cells treated with a lower dose of zVAD (5 µm), particularly when combined with GSK’872 (Figure S12M,N,Supporting Information). This finding suggests that partial inhibition of cell death enhances IAV replication. We next examined the expression of inflammatory cytokines and found that treatment with suboptimal doses of zVAD plus GSK’872 resulted in elevated levels of *IL6*, *TNF*, and *IL1B*, as well as increased expression of *IFNB* and *IFIT1* (Figure S12O–S, Supporting Information), suggesting that partial inhibition of cell death can amplify IAV‐induced inflammatory responses. This phenotype was further corroborated in vivo (Figure 6K), where mice infected with IAV and treated with sub‐optimal zVAD and GSK’872 showed higher levels of IAV NP vRNA and increased expression of *Il6*, *Il1b*, *Cxcl1*, *Cxcl5, Ifnb*, and *Ifit1* (Figure 6L–P; Figure S12T,U, Supporting Information).

Given the shared capacity of IAV and coronavirus to trigger ZBP1‐mediated cell death, we next investigated IAV replication and pulmonary inflammatory responses in ZBP1‐deficient (KO) mice after co‐infection of IAV and MHV at day 3 post‐IAV infection. Consistent with previous observations,^[^ ^6^, ^15^ ^]^ ZBP1 ablation significantly enhanced host susceptibility to IAV infection (Figure 6Q). Despite comparable mortality between genotypes following IAV and MHV co‐infection, virological analysis revealed paradoxical findings: while viral RNA accumulation was elevated in KO lungs compared to WT controls (Figure 6R), infectious viral titers were significantly lower in KO mice at day 3 post‐IAV infection (Figure 6S). This dissociation between viral RNA abundance and particle production suggests that ZBP1 deficiency impairs virion assembly during early in vivo infection. Notably, ZBP1 KO mice exhibited exacerbated pulmonary inflammation following IAV challenge, with co‐infection differentially modulating cytokine responses. MHV co‐infection in ZBP1‐deficient mice synergistically amplified *Il6* and *Tnf* expression (Figure 6T,U), whereas *Il1b*, *Cxcl1*, and *Cxcl5* levels remained refractory to further elevation (Figure 6V–X). Furthermore, type I interferon pathway activation was potentiated in ZBP1 KO lungs post‐IAV infection, with MHV co‐infection exhibiting additive effects on this signaling axis (Figure S12V,W, Supporting Information). These results imply that distinct innate signaling pathways may be regulated by cell death mediators.

In sum, these data suggest that partial inhibition of cell death by β‐coronavirus may promote IAV replication and exacerbate inflammatory responses during their co‐infection.

## 3 Discussion

**Figure 7:**
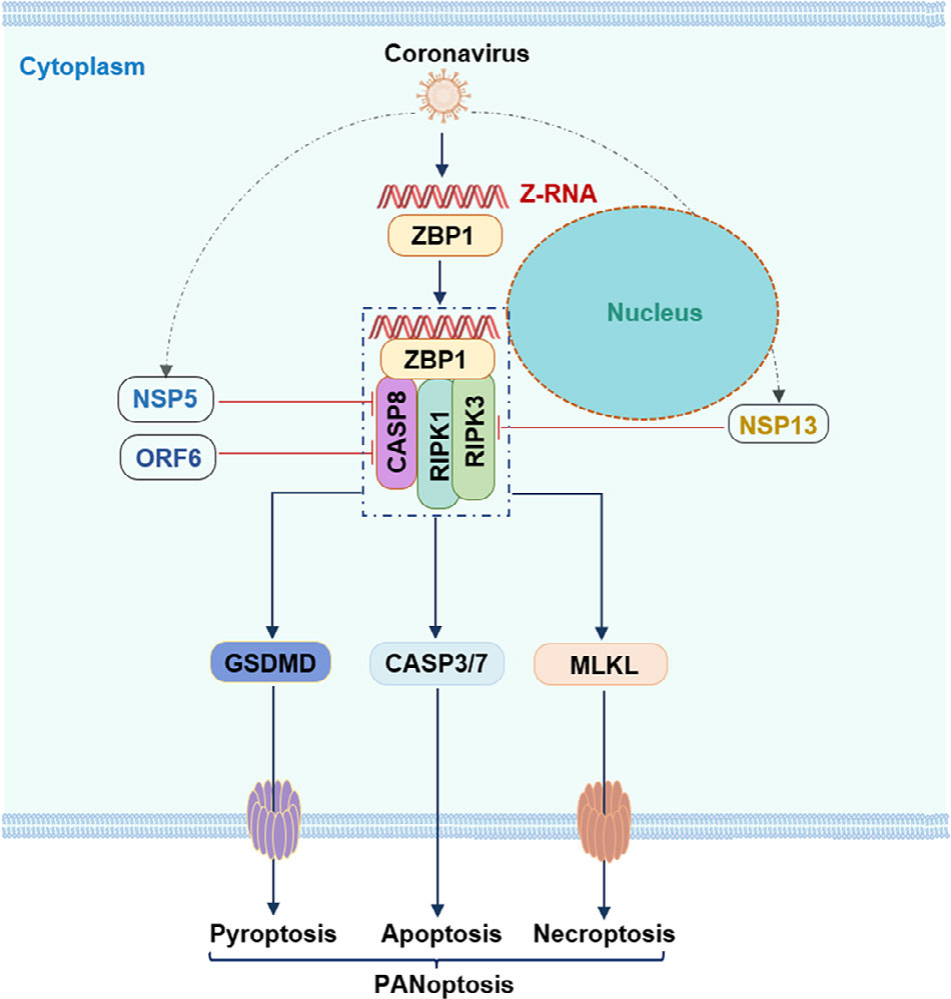
Proposed mechanism of cell death regulation by coronaviruses. During the infection, coronaviruses can produce Z‐RNA, which is subsequently recognized by ZBP1. Upon binding to Z‐RNA, ZBP1 recruits RIPK3, RIPK1, and caspase‐8, forming a large cell death complex in the cytoplasm that triggers apoptosis, pyroptosis, and necroptosis within the same cell population. To counteract the antiviral effects of programmed cell death, the viral proteins NSP5 and ORF6 interact with caspase‐8 to inhibit its activity, while NSP13 binds to RIPK3, thereby disrupting its interaction with ZBP1.

Programmed cell death is a critical mechanism in multicellular organisms for clearing infected cells to restrain viral replication and transmission. Disruption of cell death mediators has been shown to enhance viral replication in vivo.^[^ ^15^, ^16^, ^17^, ^79^, ^80^ ^]^ However, programmed cell death is not always detrimental to viruses. Both apoptotic and inflammatory caspases have been implicated in cleaving key components of the type I interferon signaling pathways, including cGAS, MAVS, and IRF3, thus inhibiting the induction of type I interferons^[^ ^70^, ^71^, ^72^ ^]^ and promoting viral replication. Additionally, influenza viruses rely on apoptotic caspases to enlarge nuclear pores to facilitate the nuclear export of viral ribonucleoprotein complexes.^[^ ^78^ ^]^ Similarly, coronaviruses require caspase‐6 to cleave their NP, which then antagonizes innate immune responses.^[^ ^24^ ^]^ Consequently, complete inhibition of apoptosis can restrict the replication of both the influenza virus and coronavirus.^[^ ^1^, ^81^, ^82^ ^]^ Therefore, modulating the host cell death mechanisms is critical for the propagation of both influenza virus and coronavirus. The NS1 protein of IAV has been reported to inhibit apoptosis.^[^ ^83^, ^84^ ^]^ However, the mechanisms by which coronaviruses regulate cell death to strike a balance between inhibition and activation during infection remain largely elusive. In this study, we demonstrated the formation of the ZBP1‐initiated cell death complex, involving Z‐RNAs, ZBP1, RIPK3, and caspase‐8, which triggers apoptosis, pyroptosis, and necroptosis, namely PANoptosis, in response to β‐coronavirus infection. We found that caspase‐8 catalytic activity is responsible for activating both apoptosis and pyroptosis in human bronchial epithelial cells during β‐coronavirus infection. To circumvent immune responses mediated by caspase‐8, SARS2 encodes NSP5 and ORF6 to modulate caspase‐8 activity. Meanwhile, NSP13 of SARS2 interacts with RIPK3 to prevent its recruitment into the ZBP1‐initiated cell death complex, thereby limiting necroptosis activation (**Figure** **7**).

While NSP5 serves as the main protease, NSP13 functions as the helicase in coronavirus, both of which are essential for coronavirus replication. Attempts to rescue SARS2 deleting either NSP5 or NSP13 were unsuccessful. Therefore, investigating the specific impact of these proteins on cell death in vivo remains challenging at this stage, necessitating the development of alternative approaches to address this question. Notably, with the exception of ORF6, which is uniquely encoded by sarbecoviruses,^[^ ^85^ ^]^ both NSP5 and NSP13 are highly conserved across various coronaviruses.^[^ ^86^, ^87^ ^]^ In this study, we have demonstrated that both NSP5 and NSP13 exert an inhibitory effect on cell death in at least two distinct β‐coronavirus species, suggesting that the suppression of cell death is a conserved function among β‐coronaviruses.

The domain of ORF6 necessary for interacting with caspase‐8 precisely aligns with that required for binding to the Rae1‐Nup98 complex^[^ ^88^ ^]^ which is essential for ORF6 suppressing interferon expression. Our in vitro cleavage assay demonstrated that ORF6 directly inhibits caspase‐8 activation, independent of other host factors. Additionally, ORF6 can disrupt the interaction between Rae1‐Nup98 and its cargo in a dose‐dependent manner in vitro,^[^ ^88^ ^]^ suggesting a direct interaction with the Rae1‐Nup98 complex. Consequently, while ORF6 binds to caspase‐8, its association with Rae1‐Nup98 complex is likely disrupted. Future investigations are imperative to elucidate how SARS2 modulates ORF6 levels to balance these dual functions. ORF6 has been shown to contribute to the pathogenicity of SARS2, as evidenced by reduced viral replication and diminished pathological lesions in mice lungs infected with ORF6‐deficient SARS2.^[^ ^89^, ^90^ ^]^ Moreover, the mortality rate is decreased in mice infected with SARS2 lacking ORF6.^[^ ^90^ ^]^ Given the dual functionality of the same domain within ORF6, further comprehensive studies are warranted to ascertain its relative contribution to SARS2 pathogenesis.

NSP13 of SARS2 has been suggested to contain an RHIM domain, which was hypothesized to compete with ZBP1 for binding to RIPK3 during SARS2 infection.^[^ ^20^, ^91^ ^]^ However, the real situation appears to be more complicated. On one hand, the RHIM mutant of SARS2‐NSP13 retains the ability to interact with RIPK3 and disrupts its interaction with ZBP1. Meanwhile, OC43‐NSP13, which possesses a dysfunctional RHIM domain^[^ ^20^ ^]^ is also capable of interacting with RIPK3 and suppressing ZBP1‐initiated necroptosis. These findings collectively suggest that NSP13 of coronaviruses inhibits ZBP1‐initiated necroptosis in a RHIM‐independent manner. On the other hand, co‐expression of NSP13 and ZBP1 has been shown to induce an unconventional form of cell death, which cannot be inhibited by zVAD plus Dabra, a finding that has been independently confirmed by another research group.^[^ ^23^ ^]^ However, overexpression of NSP13 has been shown to promote necroptosis in bat cells, suggesting functional divergence between human and bat ZBP1. Detailed studies are needed to comprehensively elucidate the effects of NSP13 on cell death in relation to its interactions with RIPK3 and ZBP1.

Although co‐infection with IAV and β‐coronaviruses has been shown to potentiate IAV replication and aggravate inflammatory pathology, the mechanistic basis underlying remains enigmatic. Given the established role of apoptotic caspases in cleaving critical innate immune regulators such as cGAS, MAVS, and IRF3,^[^ ^72^ ^]^ the increased production of inflammatory cytokines observed in IAV‐infected cells or lungs treated with a combination of zVAD and GSK’872 is likely attributed to the sustained activation of the cGAS‐STING and/or RIG‐I‐MAVS signaling pathways, which are key mediators of IAV infection‐induced immune responses.^[^ ^92^, ^93^ ^]^ Therefore, it is plausible that the enhanced IAV replication and amplified inflammatory responses in the lungs of co‐infected mice result from the ability of coronavirus‐encoded proteins to inhibit both caspase‐8 and RIPK3 activities. Our findings in ZBP1‐deficient mice further support this hypothesis, as the expression levels of certain cytokines were not further elevated in the lungs of co‐infected ZBP1‐deficient mice. The increase of other cytokines in these mice may indicate the involvement of alternative innate immune signaling pathways in the induction of those cytokines. Further research is needed to thoroughly investigate the contribution of each innate signaling pathway in mediating the inflammatory responses associated with IAV infection during the co‐infection.

Dysregulated cell death contributes to lung damage following highly pathogenic coronavirus infection; therefore, understanding the regulatory mechanisms of cell death is crucial for elucidating disease progression during coronavirus infection. While extensive research has focused on the host factors involved in the activation of cell death, less is known about how coronaviruses inhibit these processes. In this study, we demonstrate the formation of a ZBP1‐induced cell death complex during β‐coronavirus infection in human bronchial epithelial cells, which triggers the activation of PANoptosis. To counteract this, β‐coronaviruses encode several viral proteins to modulate the activation of cell death, which likely contributes to enhanced diseases during the co‐infection of IAV and β‐coronaviruses. These findings significantly advance our understanding on the regulatory mechanisms underlying coronavirus‐induced cell death, thus offering valuable perspectives for the development of therapeutic strategies aimed at mitigating lung injuries associated with highly pathogenic coronavirus infection.

## 4 Experimental Section

### Mice

The C57BL/6N mice (female, 5–6 weeks old) were purchased from the Vital River Laboratory Animal Technology Co., Ltd (Beijing) and kept in a specific pathogen‐free facility at the animal resource center at Shenzhen Bay Laboratory. Mice were maintained with a 12 h light/dark cycle and were fed standard chow. Animal studies were conducted under protocols approved by the Regional Ethics Committee for Animal Experiments at Shenzhen Bay Laboratory with the ethics number AFZM202401.

### Plasmid Constructs

Expression plasmid for SARS‐CoV‐2 spike (Addgene, 158761) was a gift from Gerald Pao,^[^ ^94^ ^]^ and expression plasmids for SARS‐CoV‐2 membrane protein (Addgene, 141386) and envelope protein (Addgene, 141385) were a gift from Nevan Krogan.^[^ ^52^ ^]^ Expression plasmids for SARS‐CoV‐2 NSP1, NSP2, NSP3N, NSP4, NSP5, NSP6, NSP7, NSP8, NSP9, NSP10, NSP12, NSP13, NSP14, NSP15, NSP16, NP, ORF3, ORF6, ORF7a, ORF7b, ORF8, ORF9c, and ORF10 cloned into pXJ2‐Flag and pcDNA6B‐Flag constructs were a gift from Peihui Wang, Shandong University. Expression plasmids for SARS‐CoV‐2 NSP3C and ORF9b were cloned into the pcDNA3.1‐N‐eGFP vector. All SARS‐CoV‐2 genes were codon optimized for human expression. Expression plasmids for 3CL‐pro of human coronavirus OC43 (hCoV‐OC43), hCoV‐229E, hCoV‐NL63, and mouse hepatitis virus (MHV) were codon optimized and synthesized by Genewiz and then cloned into the pcDNA3.1‐N‐eGFP vector.

The expression plasmids for human caspase‐8, caspase‐1, gasdermin D, and gasdermin A were a gift from Feng Shao.^[^ ^95^ ^]^ Gasdermin A with the consensus cleavage site of coronavirus 3CL‐pro was constructed by inserted the consensus sequence after the N‐terminal domain of gasdermin A.^[^ ^51^ ^]^

To obtain the catalytic dead mutants of caspase‐1, ‐3, ‐7, ‐8, NSP5, and hCoV‐OC43‐3CL‐pro, the following mutants were constructed via site mutagenesis: caspase‐1‐C285A, caspase‐3‐C163A, caspase‐7‐C186A, caspase‐8‐C360A, NSP5‐C145A, and hCoV‐OC43‐3CL‐pro‐C145A. Primers used were: caspase‐1‐C285A: 5’‐GTG ATC ATC ATC CAG GCC GCC CGT GGT GAC AGC CCT GG‐3’, 5’‐CCA GGG CTG TCA CCA CGG GCG GCC TGG ATG ATG ATC AC‐3’; caspase‐3‐C163A: 5’‐CTC TTC ATC ATT CAG GCC GCG CGG GGT ACG GAG CTG GAC‐3’, 5’‐GTC CAG CTC CGT ACC CCG CGC GGC CTG AAT GAT GAA GAG‐3’; caspase‐7‐C186A: 5’‐CCA AAC TCT TCT TCA TTC AGG CAG CGC GAG GGA CGG AGC TCG ATG ATG GAA TCC‐3’, 5’‐GGA TTC CAT CAT CGA GCT CCG TCC CTC GCG C TG CCT GAA TGA AGA AGA GTT TGG‐3’; caspase‐8‐C360A: 5’‐GTG TTT TTT ATT CAG GCT GCT CAG GGG GAT AAC TAC CAG‐3’, 5’‐CTG GTA GTT ATC CCC CTG AGC AGC CTG AAT AAA AAA CAC‐3’; NSP5‐C145A: 5’‐GTT TTC TGA ATG GTT CCG CCG GCT CTG TCG GTT TTA AC‐3’, 5’‐GTT AAA ACC GAC AGA GCC GGC GGA ACC ATT CAG AAA AC‐3’; hCoV‐OC43‐3CL‐pro‐C145A: 5’‐GCT TCC TGT GCG GCA GCG CCG GCA GCG TGG GCT AC‐3’, 5’‐GTA GCC CAC GCT GCC GGC GCT GCC GCA CAG GAA GC‐3’. To construct SARS‐CoV‐2 NSP13‐delRHIM, the following primers were used: 5’‐GAC AAA GAA TTC CAA GGA GTA CAC CTT CGA G‐3’, 5’‐CTC GAA GGT GTA CTC CTT GGA ATT CTT TGT C‐3’.

### Cell Culture and Transfection

HEK293T and HeLa cells were kind gifts from Gong Cheng, Tsinghua University, and cultivated in DMEM (Thermo Fisher Scientific, C11995500BT) supplemented with 10% fetal bovine serum (FBS) (NewZerum, FBS‐E500) and 1% penicillin and streptomycin (Sangon Biotech, E607011‐0100). HEK293 cells were a kind gift from Peihui Wang, Shandong University, and cultivated in DMEM supplemented with 10% FBS and 1% penicillin and streptomycin. Beas‐2B cells were obtained from Procell (CL‐0496) and cultured in DMEM supplemented with 10% FBS plus 1% penicillin and streptomycin. HCT‐8 cells were a kind gift from Yongping Lin, Guangzhou Medical University, and cultured in DMEM supplemented with 10% FBS and 1% penicillin and streptomycin. HT29 cells were obtained from Shanghai Cell Bank (SCSP‐5032) and cultured in DMEM supplemented with 10% FBS plus 1% penicillin and streptomycin. Vero‐E6 cells were a kind gift from Yang Liu, Shenzhen Bay Laboratory, and cultivated in DMEM supplemented with 10% FBS and 1% penicillin and streptomycin. MDCK cells were a kind gift from Wenjun Song, Guangzhou Laboratory, and cultured in MEM (Thermo Fisher Scientific, C11095500BT) plus 10% FBS and 1% penicillin and streptomycin. All cells are tested to ensure they are mycoplasma‐free prior to the experiments. For transfection assays, cells were transfected with the indicated plasmids using Hieff Trans (Yeasen, 40808ES03) according to the manufacturer's instructions. For screening the viral proteins inhibiting caspase‐8 activation, 50 ng of caspase‐8 was transfected with 200 ng of the indicated SARS‐CoV‐2 expression plasmids, respectively. Cells were then incubated for ≈20 h before sample processing unless otherwise indicated.

### CRISPR/Cas9‐Mediated Gene Deletion in Beas‐2B Cells

PX458 plasmids expressing two different gRNAs targeting caspase‐8 or ZBP1 were transfected into Beas‐2B cells. Two days post‐transfection, GFP‐positive cells were sorted via flow cytometry. Single‐cell colonies were picked then. Caspase‐8 or ZBP1 deletion was confirmed via Western blots.

### Generation of Stable Cell Lines

For inducible ZBP1 expression, GFP‐tagged ZBP1 was cloned into the vector pCW57.1. For constitutively expressing RIPK3, Myc‐tagged RIPK3 was inserted into the vector lentiCas9‐Blast (Addgene, 52962) by replacing the Cas9 gene. Plasmids for constitutively expressing E with ORF3 and ACE2 with TMPRSS2 were a kind gift from Yang Liu,^[^ ^30^ ^]^ Shenzhen Bay Laboratory. For constitutively expressing caspase‐8, Myc‐tagged caspase‐8 was cloned into the pLVX‐hygromycin vector. For expression of inducible NSP6, NSP13, and NSP14, the corresponding genes were cloned into the pLVX‐TetOne‐blasticidin vector. Lentiviruses were rescued as previously described.^[^ ^54^ ^]^ Briefly, plasmids carrying the target genes were co‐transfected with the packaging plasmids psPAX2 and pMD2.G into HEK293T cells. Lentiviruses were collected twice at days 2 and 3 after transfection. HEK293, Beas‐2B, Vero‐E6, and HT29 cells were then transduced with the indicated lentiviruses in the presence of 8 µg mL^−1^ of polybrene (Sigma, 107689) and selected via the corresponding selection markers for at least one week.

### SARS‐CoV‐2, hCoV‐OC43, Mouse Hepatitis Virus, and Influenza A Virus Culture

SARS‐CoV‐2 (USA‐WA1/2020) lacking the E and ORF3 genes but containing a mNeonGreen gene (SARS‐CoV‐2‐mNeon) was rescued as previously described.^[^ ^30^ ^]^ The same method was applied to rescue the SARS‐CoV‐2 (USA‐WA1/2020) lacking the E and ORF3 genes without a mNeonGreen gene (SARS‐CoV‐2) and SARS‐CoV‐2 (USA‐WA1/2020) lacking the E and ORF3 genes but containing a mNeonGreen gene and Flag‐tagged ORF6 (SARS‐CoV‐2‐ORF6‐Flag). The viruses were propagated and titrated in Vero‐E6 cells constitutively expressing E plus ORF3 and human ACE2 plus TMPRSS2 at 35 °C. hCoV‐OC43 was amplified in HCT‐8 cells by infecting the cells at an MOI of 0.1 at 35 °C for three days. Then the virus was titrated in Vero‐E6 cells. Mouse hepatitis virus (MHV) strain A59 was amplified as previously described.^[^ ^96^ ^]^ Viral titer was determined in 17CL‐1 cells via plaque assay. Influenza A virus A/WSN/33 (WSN) and A/PR8/34 (PR8) were rescued by reverse genetics as previously described.^[^ ^97^ ^]^ Subsequently, the virus was propagated and titrated in MDCK cells.

### Cell Infection

For hCoV‐OC43 infection, cells were infected at an MOI of 10 for cell death analysis or 5 for RT‐qPCR analysis in MEM plain media at 35 °C. After 1 h incubation, cells were supplemented with 10% FBS together with 50 ng mL^−1^ interferon α2b (IFN‐α2b) (AbMole, M10011) or 20 µm Z‐IETD‐FMK (AbMole, M3136) if indicated. Samples were collected at the indicated time points.

For SARS‐CoV‐2 infection, cells were infected at an MOI of 0.1 in MEM plain media at 35 °C. One hour later, cells were supplemented with 10% FBS together with 20 µm Z‐IETD‐FMK if indicated. Samples were collected at the indicated time points.

All the cell infections with IAV were performed using WSN. For infection of HEK293 cells stably expressing ZBP1 and RIPK3, cells were transfected with 500 ng SARS‐CoV‐2 NSP5, 500 ng hCoV‐OC43 3CL‐pro, or 250 ng SARS‐CoV‐2 ORF6 per 24‐well for 20 h and then infected with WSN at an MOI of 10 in MEM plain media at 37 °C. One hour after absorption, cells were supplemented with 10% FBS together with doxycycline (Dox) (Selleck, S4163) if indicated, and samples were collected at 20 h post‐infection. For IAV induced necroptosis, HT29 cells were infected with WSN at an MOI of 5 in MEM plain media at 37 °C. One hour after absorption, cells were supplemented with 10% FBS together with or without 500 ng mL^−1^ Dox plus 20 µm zVAD (Selleck, S7023) and 100 ng mL^−1^ IFN‐α2b. Samples were collected at 20 h post‐treatment. For gene expression analysis, Beas‐2B cells were infected with WSN at an MOI of 0.5. After one single cycle replication (≈8 h), the supernatants were removed, and zVAD plus GSK’872 (Selleck, S8465) or hCoV‐OC43 were added into the cells. The samples were collected at 24 h post‐WSN infection.

### Real‐Time Cell Death Analysis

Real‐time cell death analysis was conducted with the IncuCyte S3 imaging system (Sartorius) as described previously.^[^ ^5^ ^]^ Briefly, Cells were seeded in 24‐well plates and infected with the indicated viruses. After one hour incubation, cells were supplemented with 10% FBS together with 200 ng mL^−1^ propidium iodide (Life Technologies, P3536). The images were analyzed using IncuCyte S3 software.

### Real‐Time PCR Analysis

RNA was extracted with RNAiso Plus (Takara, 9109). cDNA was synthesized by using the PrimeScript RT reagent Kit with gDNA Eraser (Takara, RR047A) according to the manufacturer's instructions. Real‐time PCR was performed with TB Green Premix Ex Taq (Tli RNase H Plus) (Takara, RR420A) on a Bio‐Rad CFX96 RT‐PCR instrument. Primers used were as follows:

**Table:** 

18s rRNA‐F	5’‐TGT GCC GCT AGA GGT GAA ATT‐3’
18s rRNA‐R	5’‐TGG CAA ATG CTT TCG CTT T‐3’
Ifnb‐F	5’‐GCC TTT GCC ATC CAA GAG ATG C‐3’
Ifnb‐R	5’‐ACA CTG TCT GCT GGT GGA GTT C‐3’
Isg15‐F	5’‐AGC AAT GGC CTG GGA CCT AAA‐3’
Isg15‐R	5’‐AGC CGG CAC ACC AAT CTT‐3’
Ifit1‐F	5’‐CTG AGA TGT CAC TTC ACA TGG AA‐3’
Ifit1‐R	5’‐GTG CAT CCC CAA TGG GTT CT‐3’
Il6‐F	5’‐GAC AAA GCC AGA GTC CTT CAG AGA G‐3’
Il6‐R	5’‐CTA GGT TTG CCG AGT AGA TCT C‐3’
Tnf‐F	5’‐CAT CTT CTC AAA ATT CGA GTG ACA A‐3’
Tnf‐R	5’‐TGG GAG TAG ACA AGG TAC AAC CC‐3’
Il1b‐F	5’‐GAA ATG CCA CCT TTT GAC AGT G‐3’
Il1b‐R	5’‐TGG ATG CTC TCA TCA GGA CAG‐3’
Cxcl1‐F	5’‐CTG GGA TTC ACC TCA AGA ACA TC‐3’
Cxcl1‐R	5’‐CAG GGT CAA GGC AAG CCT C‐3’
Cxcl5‐F	5’‐GTT CCA TCT CGC CAT TCA TGC‐3’
Cxcl5‐R	5’‐GCG GCT ATG ACT GAG GAA GG‐3’
ACTIN‐F	5’‐CAT GTA CGT TGC TAT CCA GGC‐3’
ACTIN‐R	5’‐CTC CTT AAT GTC ACG CAC GAT‐3’
IFNB‐F	5’‐CAA CTT GCT TGG ATT CCT ACA AAG‐3’
IFNB‐R	5’‐TAT TCA AGC CTC CCA TTC AAT TG‐3’
ISG15‐F	5’‐CGC AGA TCA CCC AGA AGA TCG‐3’
ISG15‐R	5’‐TTC GTC GCA TTT GTC CAC CA‐3’
IFIT1‐F	5’‐GCG CTG GGT ATG CGA TCT C‐3’

**Table:** 

IFIT1‐R	5’‐CAG CCT GCC TTA GGG GAA G‐3’
IL6‐F	5’‐GCC TTC GGT CCA GTT GCC TT‐3’
IL6‐R	5’‐GCA GAA TGA GAT GAG TTG TC‐3’
TNF‐F	5’‐CCT CTC TCT AAT CAG CCC TCT G‐3’
TNF‐R	5’‐GAG GAC CTG GGA GTA GAT GAG‐3’
IL1B‐F	5’‐CCA CAG ACC TTC CAG GAG AAT G‐3’
IL1B‐R	5’‐GTG CAG TTC AGT GAT CGT ACA GG‐3’
IAV NP‐F	5’‐CTC GTC GCT TAT GAC AAA GAA G‐3’
IAV NP‐R	5’‐AGA TCA TCA TGT GAG TCA GAC‐3’
OC43 NP‐F	5’‐CAG GAC CGC ATG CTA AAG AC‐3’
OC43 NP‐R	5’‐TGC GCG AAG TAG ATC TGG AA‐3’
MHV M‐F	5’‐GTC TGA CTT GCC CGC TTA TG‐3’
MHV M‐R	5’‐TTC TCA ACA ATG CGG TGT CC‐3’

### Recombinant Protein Expression and Purification

The *E.coli* BL21 (DE3) (Biomed, BC201) strain was used for expressing all the recombinant proteins. For GST‐tagged proteins, protein expression was induced at 16 °C for 24 h with 0.2 mm isopropyl β‐D‐1‐thiogalactopyranoside (IPTG) (Sangon Biotech, A600168‐0005) after OD_600_ reached 0.6. Then, bacteria were collected by centrifugation, and the pellet was lysed in lysis buffer containing 1% Triton X‐100 and 5 mm DTT with mild sonication at 4 °C for 15 min, followed by centrifugation at 12 000 g for 45 min at 4 °C to clear the lysates. Subsequently, the cleared lysates were incubated with Glutathione Resin beads (GenScript, L00206) at room temperature for 1 h with rotation, after which the beads were washed three times with lysis buffer. After that, GST‐tagged proteins, including GST‐GSDMD, GST‐caspase‐3‐C/A, GST‐NSP5‐C/A, GST‐ORF6‐N, GST‐ORF6‐C, GST‐ORF6‐43‐51, and GST‐ORF6‐52‐61, on beads were eluted with elution buffer (50 mm Tris‐HCI pH 8.0, 10 mm glutathione). In the end, the proteins were stored in PBS by replacing the elution buffer with PBS using centrifugal filter units (Millipore, UFC801096).

His‐tagged caspase‐8‐C/A, caspase‐8 p18, and caspase‐8 p10 were expressed using the vector pET‐28a. The proteins were induced with 0.4 mm IPTG at 37 °C for 10 h. Bacteria harvested were resuspended and lysed in lysis buffer (50 mm Tris‐HCI pH 8.0, 150 mm NaCl, and 5 mm DTT) by sonication. Inclusion bodies, obtained by centrifugation of the lysates at 12 000 g for 1 h, were washed twice with PBS containing 1% Triton X‐100 and 5 mm DTT. The washed inclusion bodies were solubilized by stirring in 7.5 mL of the LE buffer containing 100 mm sodium dihydrogen phosphate (NaH_2_PO4), 10 mm Tris‐HCI pH 8.0, and 8 m Urea for 1 h at room temperature. The lysates were cleared by centrifugation at 12 000 g for 45 min at 4 °C. Then, cleared lysates were incubated with High Affinity Ni‐NTA Resin (GenScript, L00250) at room temperature for 1 h with rotation. Samples were washed twice with washing buffer containing 100 mm NaH_2_PO_4_, 10 mm Tris‐HCI pH 8.0, 10 mm imidazole, and 8 m Urea. Proteins on beads were then eluted with elution buffer (100 mm NaH_2_PO_4_, 10 mm Tris‐HCI pH 8.0, 300 mm imidazole, and 8 m Urea). At last, the buffer was exchanged to PBS using centrifugal filter units. Levels and integrity of purified GST or His‐tagged proteins were verified by Coomassie blue staining.

For active caspase‐8 p10/p18 tetramers, cDNAs encoding the large (p18) and small (p10) subunits were cloned into the vector pGEX‐4T‐1, respectively. Both of them were expressed and purified similar to the purification of GST‐GSDMD except that the proteins were induced with 0.2 mm IPTG. GST was removed by overnight digestion with Thrombin (Solarbio, T8021) at 37 °C. The removed GST‐tag and uncleaved GST‐fusion proteins were cleared by flowing through Glutathione Resin beads. The active p18/p10 tetramers were obtained by gently stirring the non‐tagged large and small subunits in the binding buffer (100 mm HEPES pH 7.5, 100 mm NaCl) at room temperature overnight. The supernatants were concentrated in PBS using centrifugal filter units and aliquoted for further use.

### In Vitro Cleavage by Recombinant p10/p18 Tetramers

For NSP5 inhibiting caspase‐8 p10/p18 tetramers activity, 2 µg of purified GST‐GSDMD or GST‐caspase‐3‐C/A were incubated with 0.5 µg of p10/p18 in the presence of 1 or 2 µg of NSP5, NSP5‐C/A or GST in the reaction buffer containing 50 mM HEPES pH 7.5, 3 mm EDTA, 150 mm NaCl, 0.005% (vol/vol) Tween‐20, and 10 mm DTT at 37 °C for ≈1 h. The samples were analyzed by Coomassie blue staining.

For ORF6 peptides inhibiting caspase‐8 p10/p18 tetramers activity, 2 µg of purified GST‐GSDMD or GST‐caspase‐3‐C/A were incubated with 0.5 µg of p10/p18 in the presence of 1, 3, or 5 µg of ORF6 peptides in the reaction buffer for 1 h at 37 °C unless otherwise indicated. Samples were examined by Coomassie blue staining on the SDS‐PAGE gel.

### Co‐Immunoprecipitation and GST‐Pull Down Assays

For the co‐immunoprecipitation assay, 1 µg of each plasmid was used to co‐transfect HEK293T cells seeded in 6‐well plates for 48 h unless otherwise indicated. As for those HT29 stable cell lines, cells were treated with doxycycline plus zVAD for 20 h. The Beas‐2B cells were infected with hCoV‐OC43 at an MOI of 10 for 24 h or with SARS2 at an MOI of 0.1 for the indicated times. Then cells were collected and washed once with cold PBS, following which the cells were lysed with NP‐40 lysis buffer (1% NP‐40, 150 mm NaCl, 50 mm HEPES pH 7.5). Twenty minutes later, cell lysates were centrifuged at 13 000 g for 10 min. Supernatants were transferred to new tubes and incubated overnight with 1 µg of the indicated primary antibodies on a rocking platform at 4 °C. After that, protein A/G Agarose Resin (Yeasen, 36403ES) was added and incubated for 1 h at 4 °C. The Agarose Resin was collected by centrifugation after washing 5 times with the cell lysis buffer. In the end, samples were harvested after being boiled in 2× SDS loading buffer at 100 °C for 5 min.

For GST‐pull down assay, purified 1 µg of GST or GST‐tagged proteins were incubated with 1 µg of His tagged caspase‐8‐C/A, caspase‐8 p18, or p10 in 250 µL NP‐40 lysis buffer for 4 h at 4 °C. Then, Glutathione Resin beads were added and incubated at 4 °C overnight. The Glutathione Resin beads were washed six times at room temperature for 5 min with the washing buffer (1% NP‐40, 500 mm NaCI, 50 mm HEPES pH 7.5) and then the beads were resuspended in 2× SDS loading buffer and boiled at 100 °C for 5 min.

### Immunofluorescence Microscopy

Cells were infected by the indicated viruses or co‐transfected with the indicated plasmids for 20 h. Then the cells were washed once with cold PBS and fixed in freshly prepared 4% paraformaldehyde for 15 min, followed with permeabilization in PBS containing 0.5% Triton X‐100 for 10 min. After being blocked with 5% Normal Donkey Serum for 30 min, cells were incubated with primary antibodies for 1 h at 37 °C. Then cells were incubated with fluorophore‐conjugated secondary antibodies for 1 h at 37 °C. After this, slides were mounted in mounting media with DAPI‐Aqueous Fluoroshield (Abcam, ab104139). Images were acquired on a Zeiss LSM980. The antibodies used were: Z‐NA (Absolute antibody, Ab00783‐3.0, 1:200), RIPK3 (CST, 10188, 1:100), DDDDK‐tag (Abclonal, AE092, 1:100), Myc (SinoBiological, 100029‐MM08, 1:200), donkey anti‐mouse IgG H&L (Alexa Flour 488) (Abcam, ab150105, 1:500), donkey anti‐mouse IgG H&L (Alexa Flour 594) (Abcam, ab150108, 1:500), donkey anti‐rabbit IgG H&L (Alexa Flour 594) (Abcam, ab150076, 1:500) and donkey anti‐rabbit IgG H&L (Alexa Flour 647) (Invitrogen, A21245, 1:500).

### Immunoblot Analysis

For caspase‐1 analysis, cells seeded in 24‐well plates were lysed with the supernatants using 30 µL caspase lysis buffer (1× protease inhibitors, 1× phosphatase inhibitors, 10% NP‐40, and 25 mM DTT). Then samples were boiled with 100 µL 4× SDS loading buffer. For analysis of IL‐1β expression, the supernatants and cells were collected separately and lysed with caspase lysis buffer and RIPA lysis buffer, respectively. For analysis of the other proteins, RIPA lysis buffer was used to lyse the cells, while the supernatants were discarded.

Electrophoresis was performed via 8–12% polyacrylamide gels. Then, proteins were transferred onto PVDF membranes (Roche, 3010040001) and blocked with 5% skim milk for 1 h at room temperature. All the primary antibodies were incubated with the membrane overnight at 4 °C. Then, secondary antibodies with HRP were incubated at room temperature for 1 h. Images were acquired via a GE Amersham ImageQuant 800.

The antibodies used were: anti‐caspase‐8 (Enzo, ALX‐804‐242‐C100, 1:1000), anti‐caspase‐8 (AdipoGen, AG‐20T‐0138‐C100, 1:1000), anti‐cleaved caspase‐8 (CST, 8592, 1:1000), anti‐caspase‐3 (CST, 9662S, 1:1000), anti‐cleaved caspase‐3 (CST, 9661, 1:1000), anti‐caspase‐7 (CST, 9492, 1:1000), anti‐cleaved caspase‐7 (CST, 9491, 1:1000), anti‐caspase‐1 (R&D, mab6215, 1:1000), anti‐caspase‐1 (CST, 2225, 1:1000), anti‐hCoV‐OC43 nucleoprotein (SinoBiological, 40643‐T62, 1:1000), anti‐GSDMD (Abclonal, A20197, 1:1000), anti‐cleaved GSDMD (N terminal) (Abclonal, A22523, 1:1000), anti‐GSDME (Abcam, ab215191, 1:1000), anti‐cleaved IL‐1β (Abclonal, A1112, 1:1000), anti‐pro‐IL‐1β (SinoBiological, 10139‐M201, 1:1000), anti‐SARS‐CoV‐2 nucleoprotein (Abclonal, A18797, 1:1000), anti‐SARS‐CoV‐2 NSP5 (Abclonal, A20198, 1:1000), anti‐NLRP3 (AdipoGen, AG‐20B‐0014, 1:1000), anti‐ZBP1 (CST, 60968S, 1:1000), anti‐RIPK1 (CST, 3493, 1:1000), anti‐RIPK3 (CST, 10188, 1:1000), anti‐pMLKL (abcam, ab187091, 1:1000; CST, 91689S, 1:1000), anti‐MLKL (CST, 14993S, 1:1000), anti‐ASC (AdipoGen, AG‐25B‐0006‐C100, 1:1000), anti‐ACE2 (SinoBiological, 10108‐R003, 1:1000), anti‐TMPRSS2 (SinoBiological, 204314‐T08, 1:1000), anti‐influenza A virus NS1 (Santa Cruz, sc‐130568, 1:1000), anti‐Flag (Sigma, F1804, 1:5000), anti‐HA (Thermo Fisher Scientific, 206183, 1:1000), anti‐Strep (MBL, M211‐3, 1:1000), anti‐GFP (SinoBiological, 13105‐R208, 1:1000), anti‐GST (OriGene, TA150102, 1:1000), anti‐Myc (SinoBiological, 100029‐MM08, 1:1000), anti‐His (OriGene, TA150088, 1:1000), anti‐actin (Proteintech, 66009‐1‐IG, 1:5000), and HRP‐conjugated secondary antibodies (Jackson ImmunoResearch Laboratories, anti‐rabbit [111‐035‐047], 1:5000; anti‐mouse [315‐035‐047], 1:5000).

### Animal Infection

Age‐ and sex‐matched wild‐type mice, 6‐ to 8‐week‐old, were anesthetized with 250 mg kg^−1^ Avertin and then infected intranasally with PR8 or MHV in 50 µL PBS as indicated. For PR8 infection, each mouse received ≈100 plaque‐forming units (PFU) of PR8, and for MHV infection, each mouse was inoculated with ≈1000 PFU of MHV. Mice treated with zVAD and/or GSK’872 received intraperitoneal injections of 10 mg kg^−1^ of each compound. Infected mice were monitored daily over two weeks for the survival study. Mice displaying severe signs of disease or more than 30% weight loss relative to pre‐infection body weight were sacrificed. Lungs harvested at the indicated time points were homogenized in 1 mL PBS for viral titers and gene expression to be determined by plaque assays and RT‐PCR, respectively, or fixed with formalin for histological analysis. Lung sections were processed by Servicebio (Wuhan).

### Statistical Analysis

GraphPad Prism 8.0 software was used for data analysis. Data are represented as mean ± SEM. Statistical significance was determined by Student's *t‐*test (two‐tailed) for two groups, ANOVA (with Dunnett's multiple comparisons test or Turkey's multiple comparisons test) for three or more groups, or Log‐rank test for the survival curves. *P‐*values less than 0.05 were considered statistically significant, where *P < 0.05, **P < 0.01, ***P < 0.001, and ****P < 0.0001.

## Conflict of Interest

The authors declare no conflict of interest.

## Author Contributions

H.W. and M.L. contributed equally to this work. M.Z. conceptualized the study. M.Z., Y.L., and H.L.C. designed the methodology. M.Z., H.W., and M.D.L. performed the majority of the experiments. J.Z., F.F.Z., M.M.C., M. D. L., W.J.S., and F.H. made all the plasmids needed. Y.L., S.W.L., P.W., and H.T. conducted the SARS‐CoV‐2 infection experiment. M.Z., Y.L., R.K., H.W., and M.D.L. conducted the analysis. R.K., W.J.S., Y.P.L., and P.H.W. provided critical reagents and scientific discussion, M.Z. wrote the manuscript with input from all authors. M.Z. and Y.L. acquired the funding, and M.Z. provided overall supervision.

## Supporting information



Supporting Information

## Data Availability

The data that support the findings of this study are available on request from the corresponding author. The data are not publicly available due to privacy or ethical restrictions.
