# Supplementary material for: The NSP5, ORF6 and NSP13 of SARS‐CoV‐2 Cooperate to Modulate Inflammatory Cell Death Activation
Source: Adv Sci (Weinh). 2025 Aug 14;12(41):e03977. doi: 10.1002/advs.202503977 (PMC12591158; doi:10.1002/advs.202503977)
Supplement: Supplementary file 1 — Supporting Information [file ADVS-12-e03977-s001.docx]

**SUPPLEMENTAL FIGURE LEGENDS**


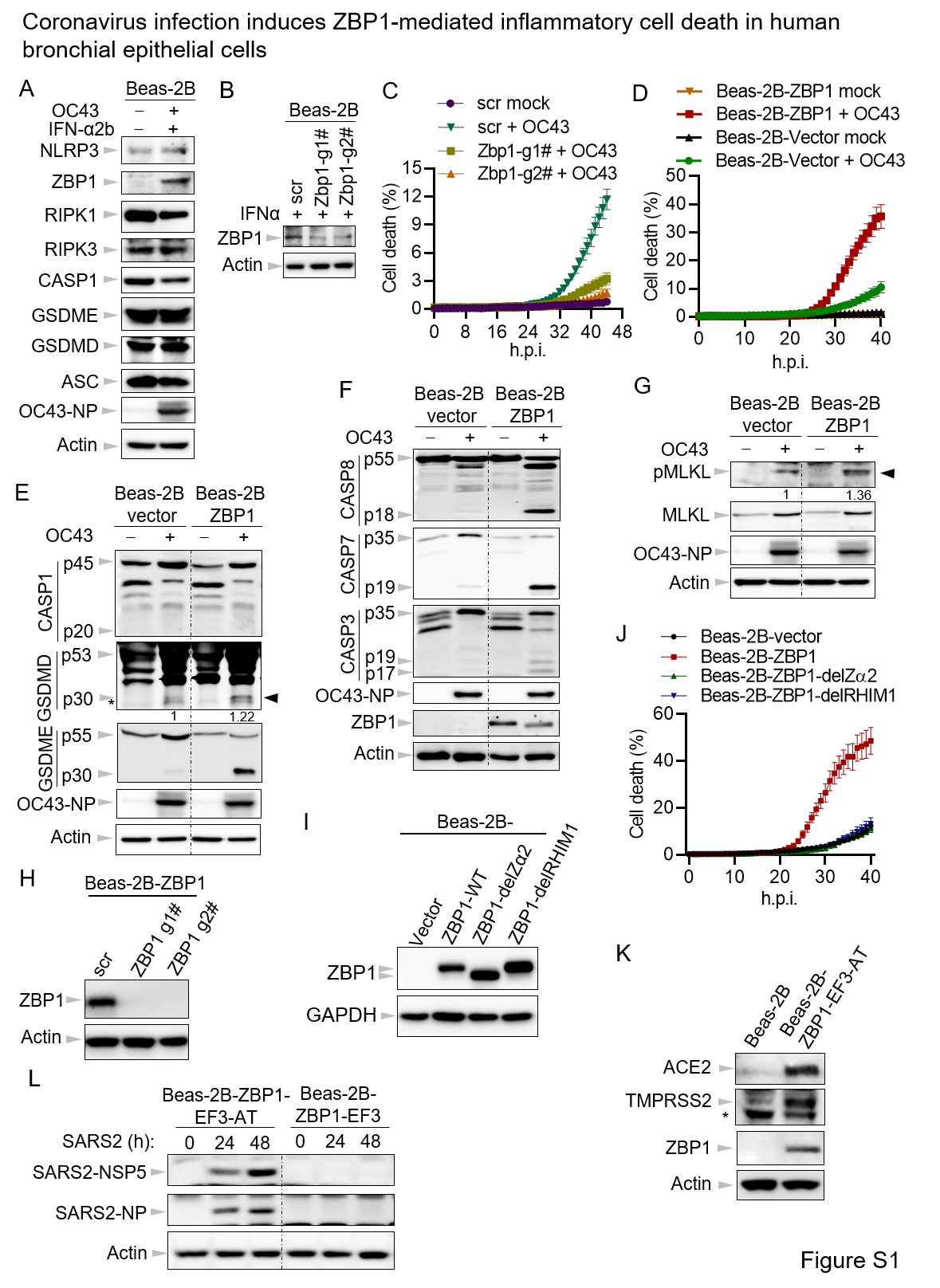


**Figure S1.** **Coronavirus infection induces ZBP1-mediated inflammatory cell death in human bronchial epithelial cells, related to Figure 1.**

(A) Immunoblot analysis of NLRP3, ZBP1, RIPK1, RIPK3, caspase-1 (CASP1), gasdermin E (GSDME), gasdermin D (GSDMD), ASC, and OC43 nucleocapsid protein (NP) in interferon-α2b (IFN-α2b) treated Beas-2B cells with or without hCoV-OC43 (OC43) infection for 30 h. Actin is used as the internal control. (B) Immunoblot analysis of ZBP1 in Beas-2B cells following CRISPR-directed deletion of ZBP1. Actin is used as the internal control. (C) Real-time analysis of cell death in Beas-2B cells following CRISPR-directed deletion of ZBP1 in response to OC43 infection. (D) Real-time analysis of cell death in OC43-infected Beas-2B-ZBP1 or Beas-2B-vector cells. (E–G) Immunoblot analysis of pro- and cleaved forms of CASP1, GSDMD, GSDME, and OC43 NP (E), pro- and cleaved forms of caspase-8 (CASP8), -3 (CASP3), -7 (CASP7), OC43 NP, and ZBP1 (F), phosphorylated mixed lineage kinase domain-like protein (pMLKL), total MLKL, and OC43 NP (G) after OC43 infection with Beas-2B-vector or Beas-2B-ZBP1 cells for 30 h. Actin is used as the internal control. (H) Immunoblot analysis of ZBP1 in Beas-2B-ZBP1 cells following CRISPR-directed deletion of ZBP1. Actin is used as the internal control. (I) Immunoblot analysis of ZBP1 in Beas-2B-vector, Beas-2B-ZBP1, Beas-2B-ZBP1-delZα2, and Beas-2B-ZBP1-delRHIM1 cells. GAPDH is used as the internal control. (J) Real-time analysis of cell death in OC43-infected Beas-2B-vector, Beas-2B-ZBP1, Beas-2B-ZBP1-delZα2, and Beas-2B-ZBP1-delRHIM1 cells. (K) Immunoblot analysis of ACE2, TMPRSS2 and ZBP1 in Beas-2B and Beas-2B-ZBP1-E-ORF3-ACE2-TMPRSS2 (Beas-2B-ZBP1-EF3-AT) cells. Actin is used as the internal control. (L) Immunoblot analysis of SARS-CoV-2-NSP5 (SARS2-NSP5) and SARS2-NP in the indicated cells infected with SARS2 for the indicated time points. Actin is used as the internal control. Asterisks designate non-specific bands. Data are shown as mean ± SEM (n = 4) (C, D, and J). Data are representative of three independent experiments.


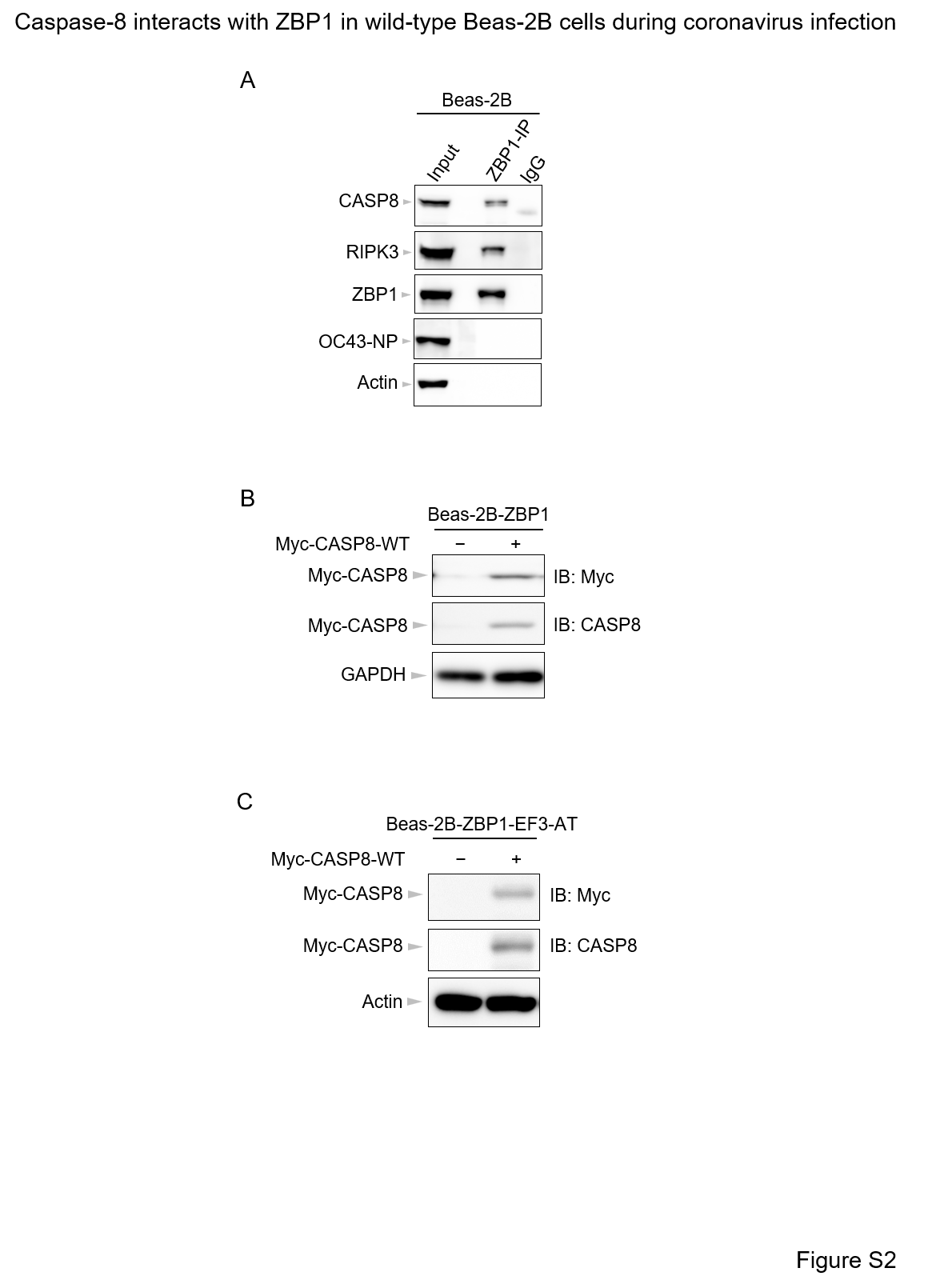


**Figure S2.** **Caspase-8 interacts with ZBP1 in wild-type Beas-2B cells during coronavirus infection, related to Figure 2.**

(A) Immunoprecipitates and total lysates from Beas-2B cells after infection of hCoV-OC43 for 24 h. (B) Immunoblot analysis of Myc-tagged caspase-8 (Myc-CASP8) in CASP8 knock-out Beas-2B-ZBP1 cells overexpressing Myc-CASP8. GAPDH is used as the internal control. (C) Immunoblot analysis of Myc-CASP8 in CASP8 knock-out Beas-2B-ZBP1-E-ORF3-ACE2-TMPRSS2 cells (Beas-2B-ZBP1-EF3-AT) overexpressing Myc-CASP8. Actin is used as the internal control. Data are representative of three independent experiments.


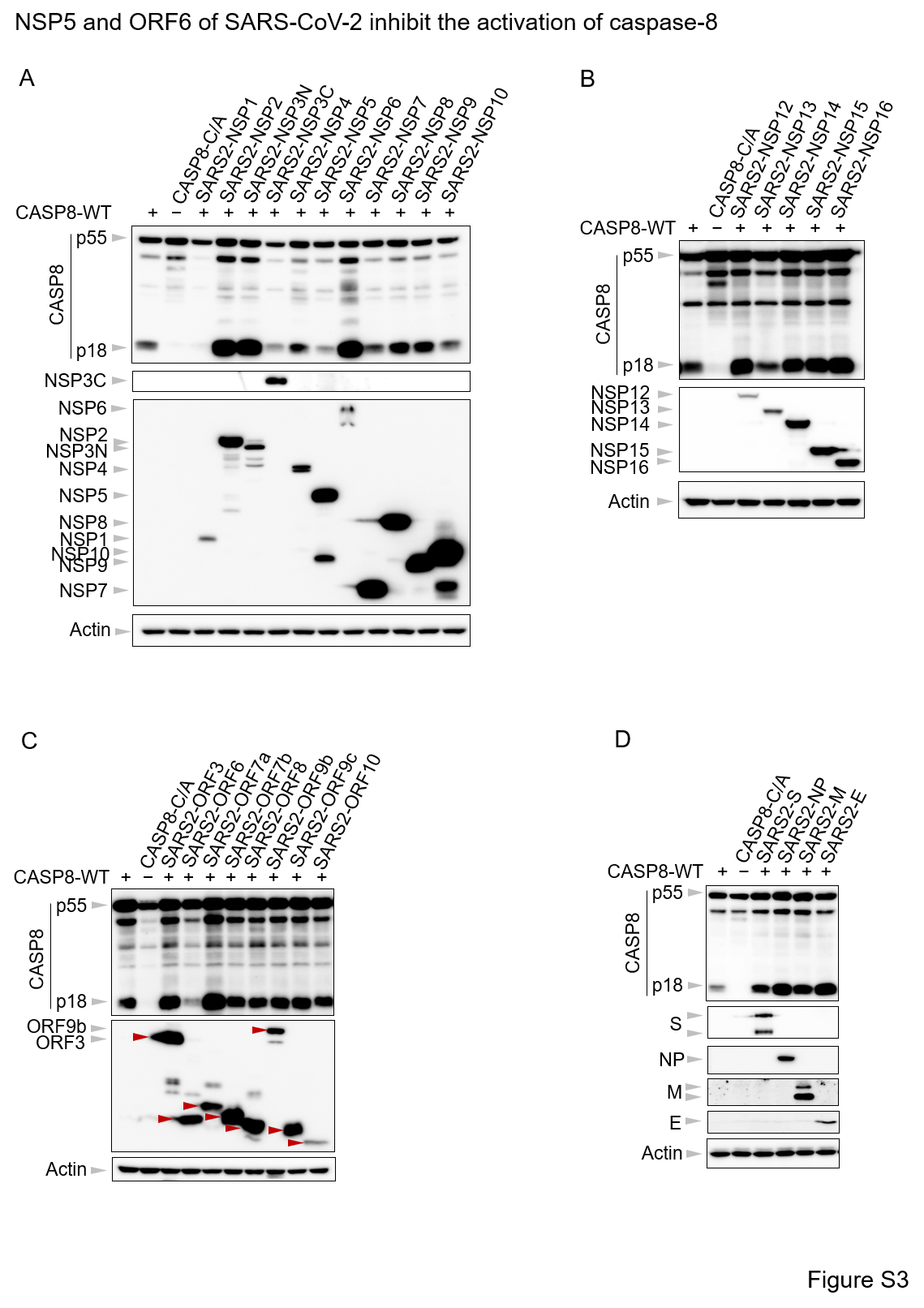


**Figure S3.** **NSP5 and ORF6 of SARS-CoV-2 inhibit the activation of caspase-8, related to Figure 2.**

(A) Immunoblot analysis of pro- and cleaved forms of caspase-8 (CASP8), non-structural protein 3 C-terminus (NSP3C), NSP6, NSP2, NSP3N, NSP4, NSP5, NSP8, NSP1, NSP10, NSP9 and NSP7 of SARS-CoV-2 (SARS2) in HEK293T cells transfected with the indicated expression plasmids. Actin is used as the internal control. NSP6 detected here is not the monomer, but the oligomerized form. (B) Immunoblot analysis of pro- and cleaved forms of CASP8, NSP12, NSP13, NSP14, NSP15, and NSP16 of SARS2 in HEK293T cells transfected with the indicated expression plasmids. Actin is used as the internal control. (C) Immunoblot analysis of pro- and cleaved forms of CASP8, open reading frame 9b (ORF9b), ORF3, ORF6, ORF7a, ORF7b, ORF8, ORF9c, and ORF10 of SARS2 in HEK293T cells transfected with the indicated expression plasmids. Actin is used as the internal control. Red arrows indicate the correct bands for each protein. (D) Immunoblot analysis of pro- and cleaved forms of CASP8, spike (S), nucleocapsid (NP), membrane (M), and envelope (E) protein of SARS2 in HEK293T cells transfected with the indicated expression plasmids. Actin is used as the internal control. Data are representative of three independent experiments.


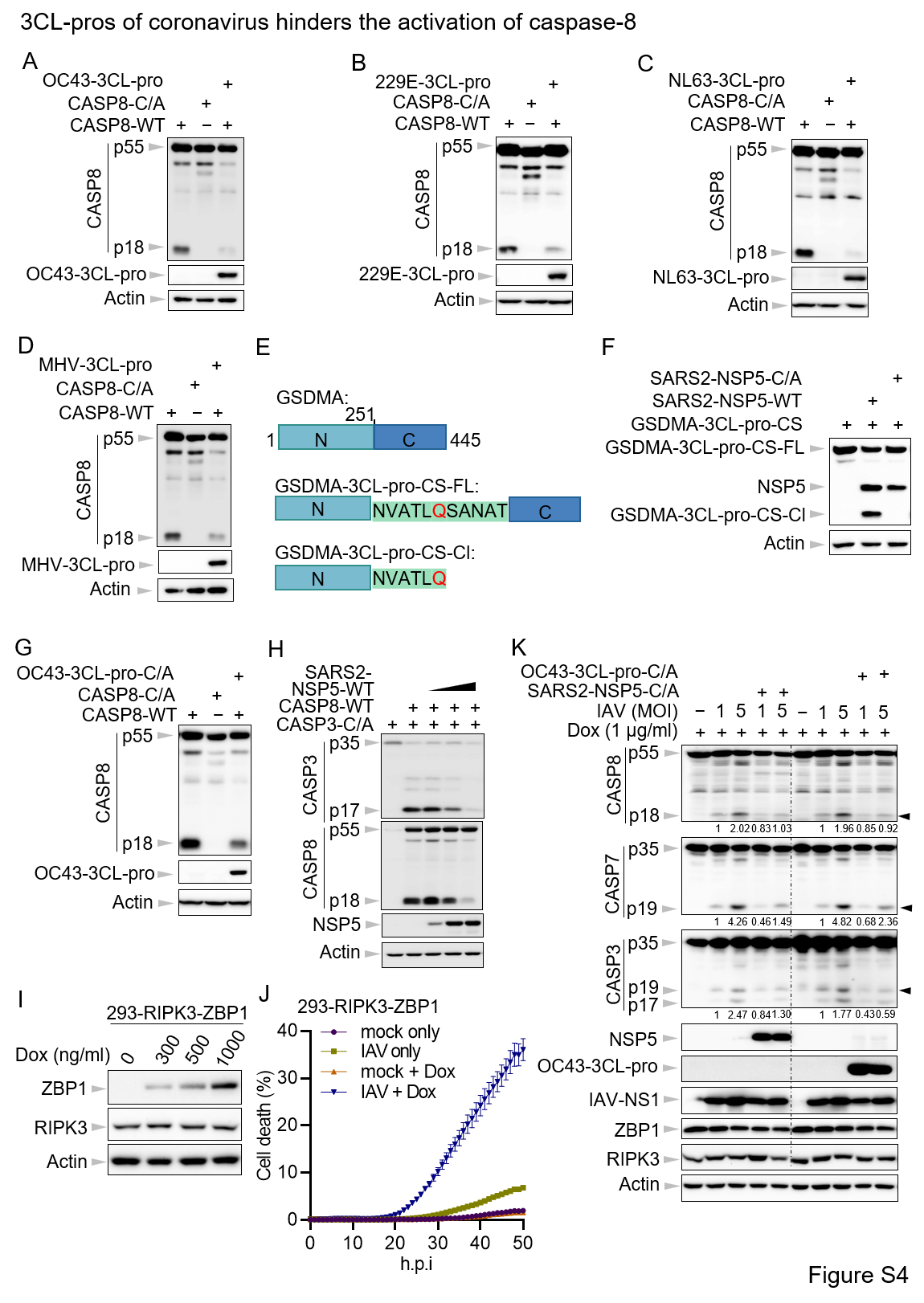


**Figure S4.** **3CL-pro of coronavirus hinders the activation of caspase-8, related to Figure 3.**

(A–D) Immunoblot analysis of pro- and cleaved forms of caspase-8 (CASP8) after co-transfection with hCoV-OC43-3CL-pro (OC43-3CL-pro) (A), hCoV-229E-3CL-pro (229E-3CL-pro) (B), hCoV-NL63-3CL-pro (NL63-3CL-pro) (C), and mouse hepatitis virus 3CL-pro (MHV-3CL-pro) (D) in HEK293T cells. Actin is used as the internal control. (E) Schematic depiction of the domains of gasdermin A (GSDMA), insertion of consensus cleavage sequence of coronavirus 3CL-pro in GSDMA (GSDMA-3CL-pro-CS-FL) and the N-terminal domain of GSDMA-3CL-pro-CS-FL after cleavage by coronavirus 3CL-pro (GSDMA-3CL-pro-CS-Cl). The red glutamine (Q) indicates the cleavage site recognized by coronavirus 3CL-pro. (F) Immunoblot analysis of pro- (FL) and cleaved (Cl) forms of GSDMA-3CL-pro-CS, and OC43-3CL-pro in HEK293T cells transfected with the indicated expression plasmids. Actin is used as the internal control. (G) Immunoblot analysis of pro- and cleaved forms of CASP8 in HEK293T cells transfected with the indicated expression plasmids. Actin is used as the internal control. (H) Immunoblot analysis of pro- and cleaved forms of caspase-3 (CASP3), CASP8 and NSP5 in HEK293T cells transfected with the indicated expression plasmids. Actin is used as the internal control. (I) Immunoblot analysis of ZBP1 and RIPK3 in 293-RIPK3-ZBP1 cells treated with increasing doses of doxycycline (Dox). Actin is used as the internal control. (J) Real-time analysis of cell death in influenza A virus (IAV) infected 293-RIPK3-ZBP1 cells with or without Dox. (K) Immunoblot analysis of pro- and cleaved forms of CASP8, caspase-7 (CASP7), CASP3, SARS2 NSP5, OC43-3CL-pro, IAV-NS1, ZBP1, and RIPK3 in IAV infected 293-RIPK3-ZBP1 cells transfected with the indicated expression plasmids. Actin is used as the internal control. Data are shown as mean ± SEM (n = 4) (J). Data are representative of three independent experiments.


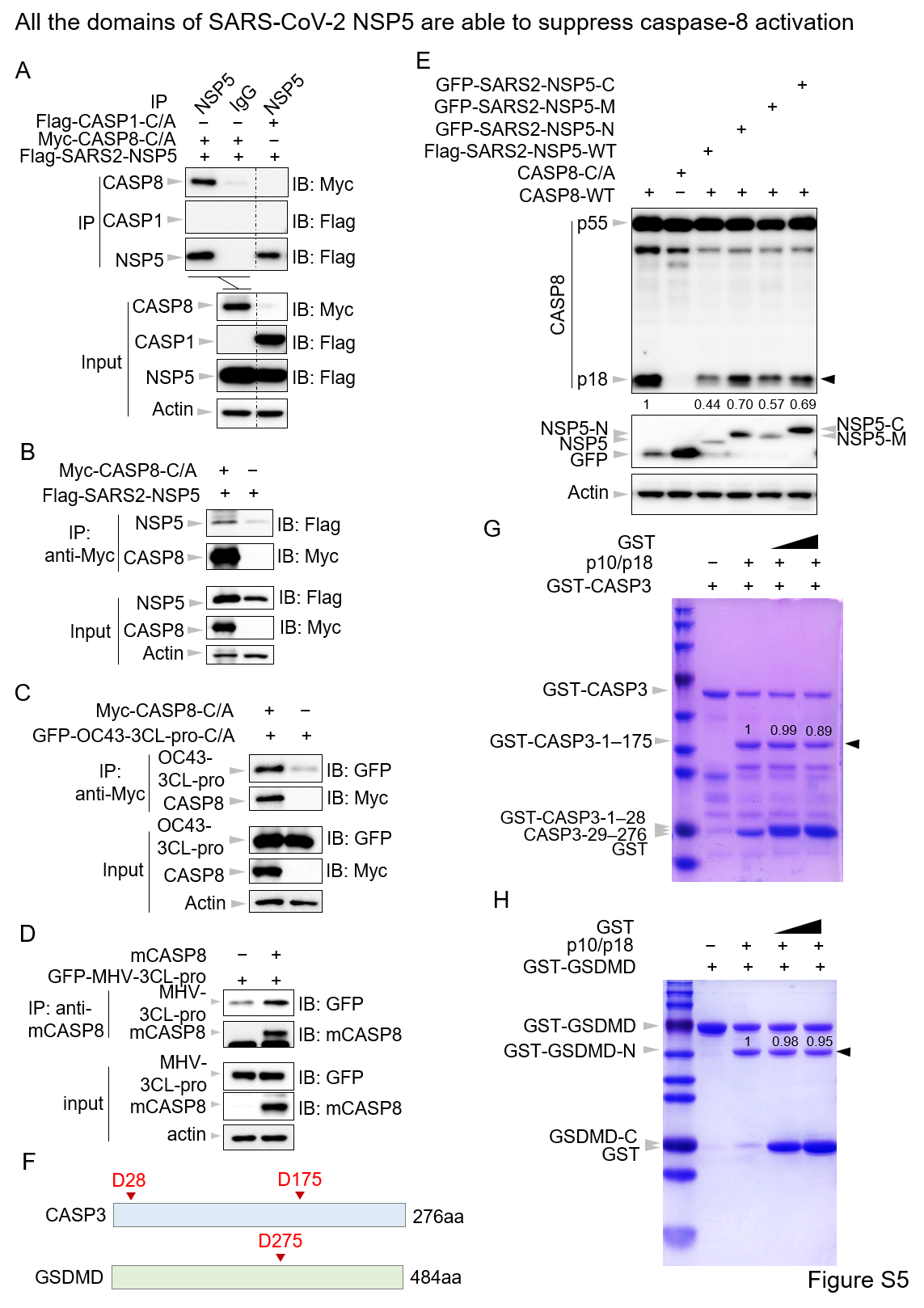


**Figure S5.** **All the domains of SARS-CoV-2 NSP5 are able to suppress caspase-8 activation, related to Figure 3.**

(A) Immunoprecipitates and total lysates from HEK293T cells after co-transfection Flag-tagged SARS-CoV-2 NSP5 (Flag-SARS2-NSP5) with Flag-tagged catalytic dead mutant of caspase-1 (Flag-CASP1-C/A) or Myc-tagged catalytic dead mutant of caspase-8 (Myc-CASP8-C/A) for 48 h. (B) Immunoprecipitates and total lysates from HEK293T cells after co-transfection of Flag-SARS2-NSP5 and Myc-CASP8-C/A for 48 h. (C) Immunoprecipitates and total lysates from HEK293T cells after co-transfection of GFP-tagged catalytic dead mutant of 3CL-pro of hCoV-OC43 (GFP-OC43-3CL-pro-C/A) and Myc-CASP8-C/A for 48 h. (D) Immunoprecipitates and total lysates from HEK293T cells after co-transfection of GFP-tagged 3CL-pro of MHV (GFP-MHV-3CL-pro) and mouse caspase-8 (Mcasp8) for 48 h. (E) Immunoblot analysis of pro- and cleaved forms of CASP8, SARS2-NSP5-WT, SARS2-NSP5-N, SARS2-NSP5-M and SARS2-NSP5-C in HEK293T cells transfected with the indicated expression plasmids. Actin is used as the internal control. (F) Schematic depiction of the cleavage sites by CASP8 in caspase-3 (CASP3) and gasdermin D (GSDMD). (G and H) In vitro cleavage of purified GST-tagged CASP3 (GST-CASP3) (G) and GSDMD (GST-GSDMD) (H) by active CASP8 p10/p18 tetramer in the presence of increasing doses of GST. Data are representative of three independent experiments.


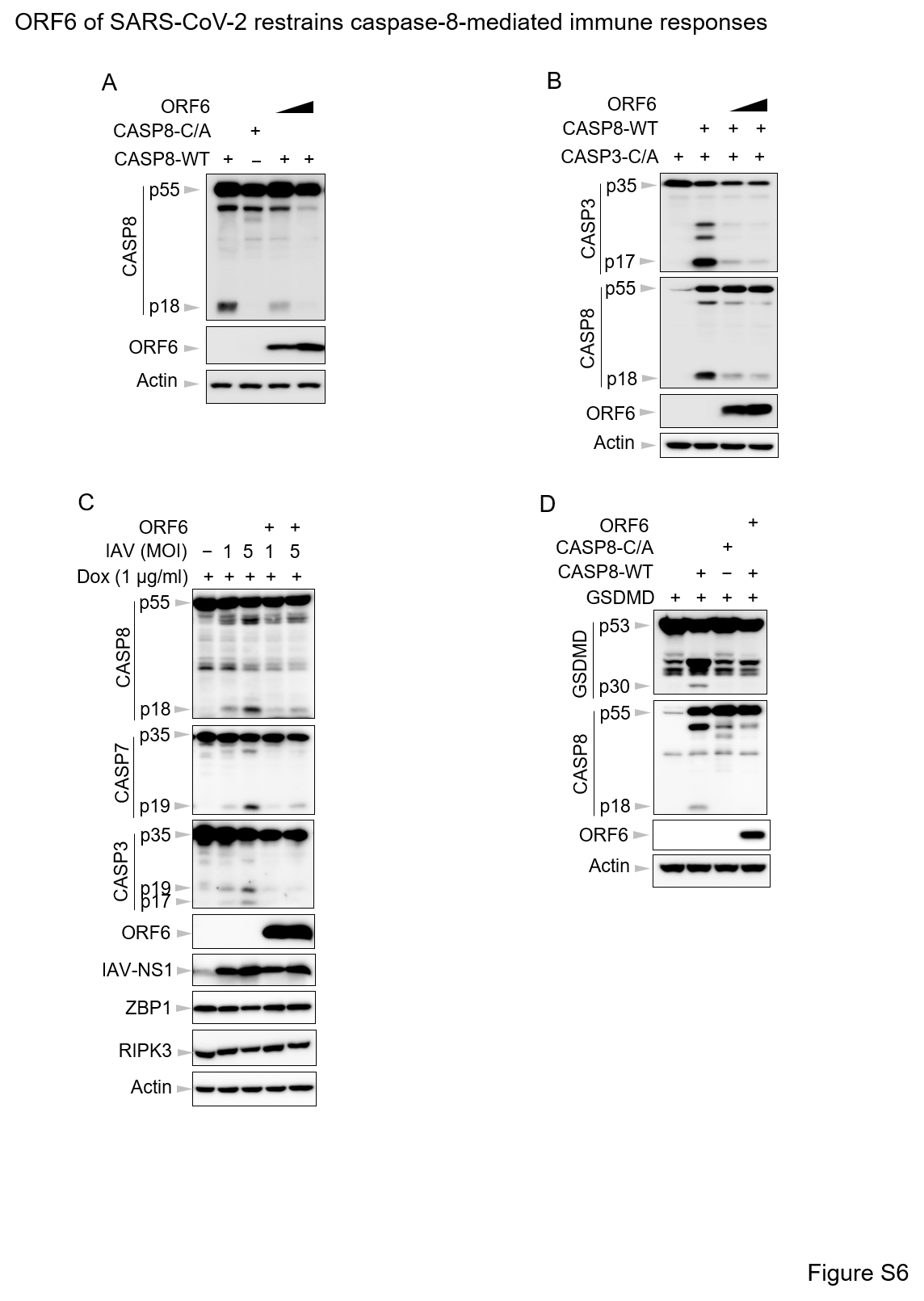


**Figure S6.** **ORF6 of SARS-CoV-2 restrains caspase-8-mediated immune responses, related to Figure 4.**

(A) Immunoblot analysis of pro- and cleaved forms of caspase-8 (CASP8) and SARS-CoV-2 (SARS2) ORF6 in HEK293T cells transfected with the indicated expression plasmids. Actin is used as an internal control. (B) Immunoblot analysis of pro- and cleaved forms of caspase-3 (CASP3), CASP8, and SARS2 ORF6 in HEK293T cells transfected with the indicated expression plasmids. Actin is used as an internal control. (C) Immunoblot analysis of pro- and cleaved forms of CASP8, caspase-7 (CASP7), CASP3, SARS2 ORF6, influenza A virus (IAV) NS1, ZBP1 and RIPK3 in doxycycline (Dox) treated IAV infected 293-RIPK3-ZBP1 cells after transfection with SARS2 ORF6. Actin is used as the internal control. (D) Immunoblot analysis of pro- and cleaved forms of gasdermin D (GSDMD), CASP8 and ORF6 in HEK293T cells transfected with the indicated expression plasmids. Actin is used as an internal control. Data are representative of three independent experiments.


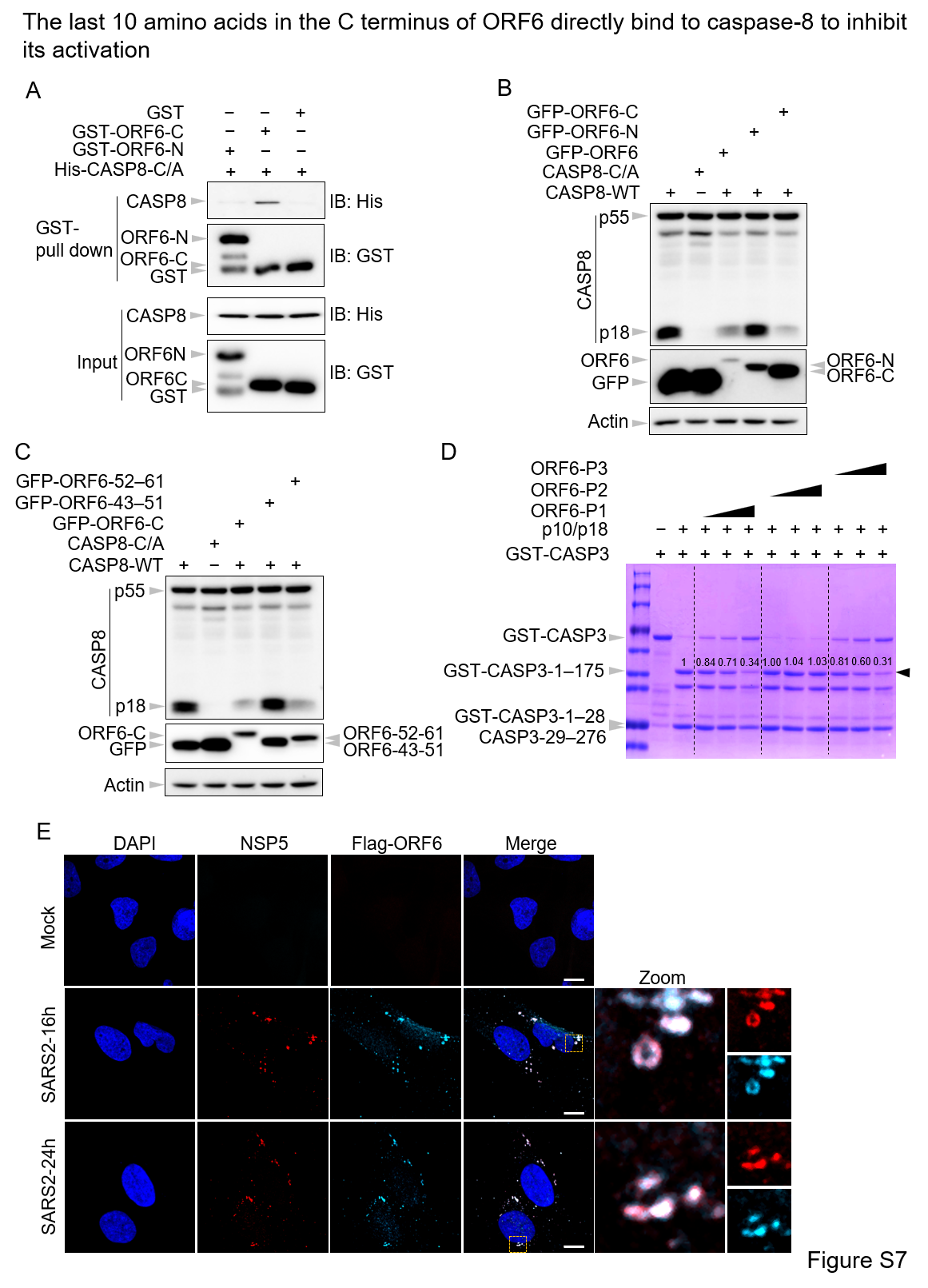


**Figure S7.** **The last 10 amino acids in the C terminus of ORF6 directly bind to caspase-8 to inhibit its activation, related to Figure 4.**

(A) GST-pull down assay for the interaction between GST-tagged ORF6-C (GST-ORF6-C) or ORF6-N (GST-ORF6-N) and His-tagged catalytic dead mutant of caspase-8 (His-CASP8-C/A). (B) Immunoblot analysis of pro- and cleaved forms of CASP8, ORF6, ORF6-N, ORF6-C, and GFP in HEK293T cells transfected with the indicated expression plasmids. Actin is used as the internal control. (C) Immunoblot analysis of pro- and cleaved forms of CASP8, ORF6-C, ORF6-43–51, ORF6-52–61, and GFP in HEK293T cells transfected with the indicated expression plasmids. Actin is used as the internal control. (D) In vitro cleavage of purified GST-tagged caspase-3 (GST-CASP3) by active CASP8 p10/p18 tetramer in the presence of increasing doses of SARS2 ORF6-P1, ORF6-P2 or ORF6-P3. (E) Confocal images of Beas-2B-ZBP1-EF3-AT cells infected with SARS2 encoding Flag-tagged ORF6. Scale bar, 10 μm. Data are representative of three independent experiments.


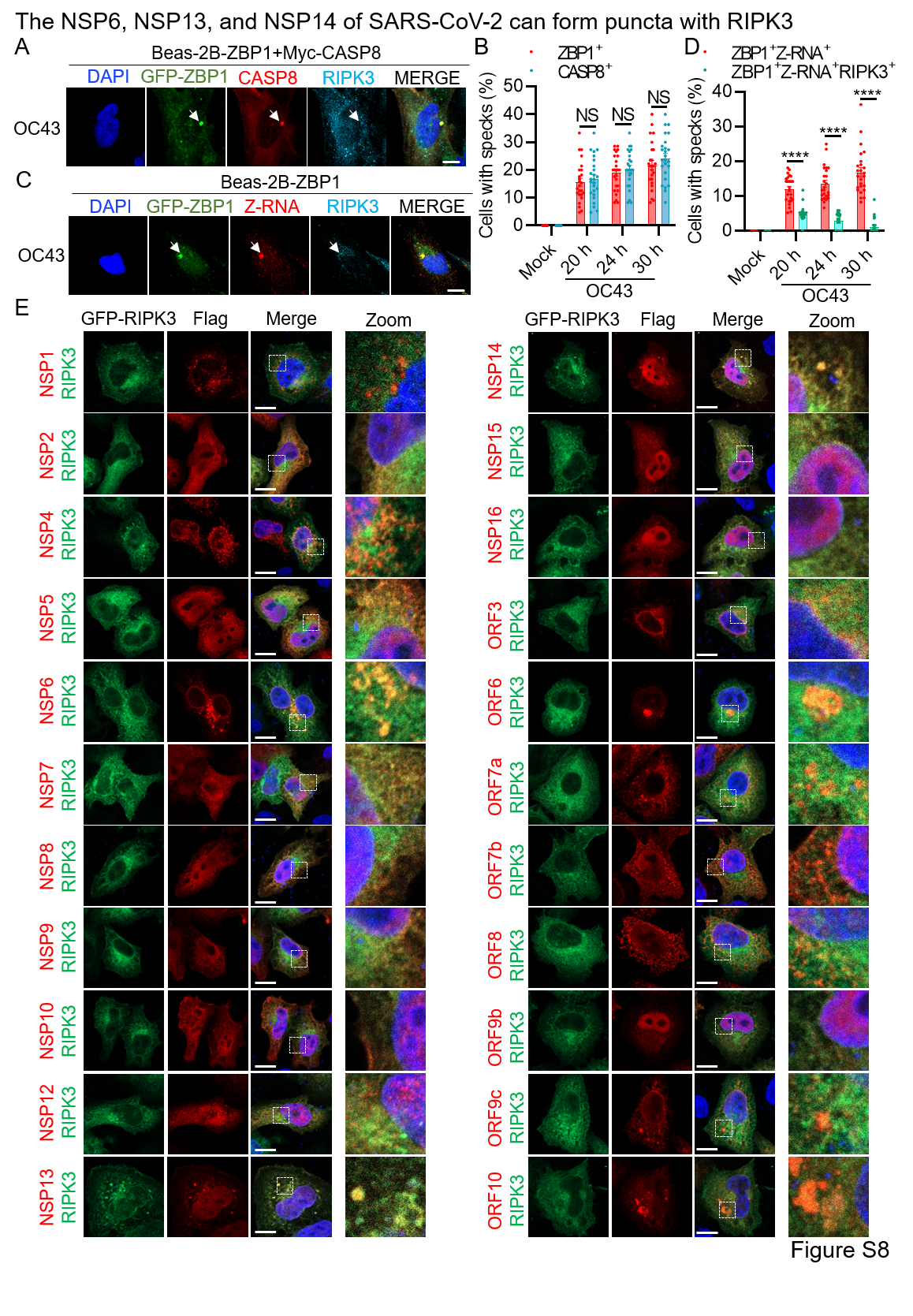


**Figure S8. The NSP6, NSP13, and NSP14 of SARS-CoV-2 can form puncta with RIPK3, related to Figure 5.**

(A) Confocal images of caspase-8 (CASP8) knock-out Beas-2B-ZBP1 cells overexpressing Myc-CASP8 (Beas-2B-ZBP1 + Myc-CASP8) after infection with hCoV-OC43 (OC43) for 24 h. Scale bar, 10 μm. (B) Quantification of the percentage of cells with ZBP1^+^ or CASP8^+^ specks in A at the indicated time points. (C) Confocal images of Beas-2B-ZBP1 cells infected with OC43 for 24 h. Scale bar, 10 μm. (D) Quantification of the percentage of cells with ZBP1^+^Z-RNA^+^ or ZBP1^+^Z-RNA^+^RIPK3^+^ specks in C at the indicated time points. (E) Confocal images of HeLa cells co-transfected with Flag-tagged SARS-CoV-2-encoded non-structural or accessory proteins and GFP-tagged RIPK3 for 20 h. Inset panels are magnified 4.5 ×. Scale bar, 10 μm. NS, not significant; *****P* < 0.0001. Analysis was performed using two-way ANOVA (B and D). Data are shown as mean ± SEM (n = 25) (B and D). Data are representative of three independent experiments.


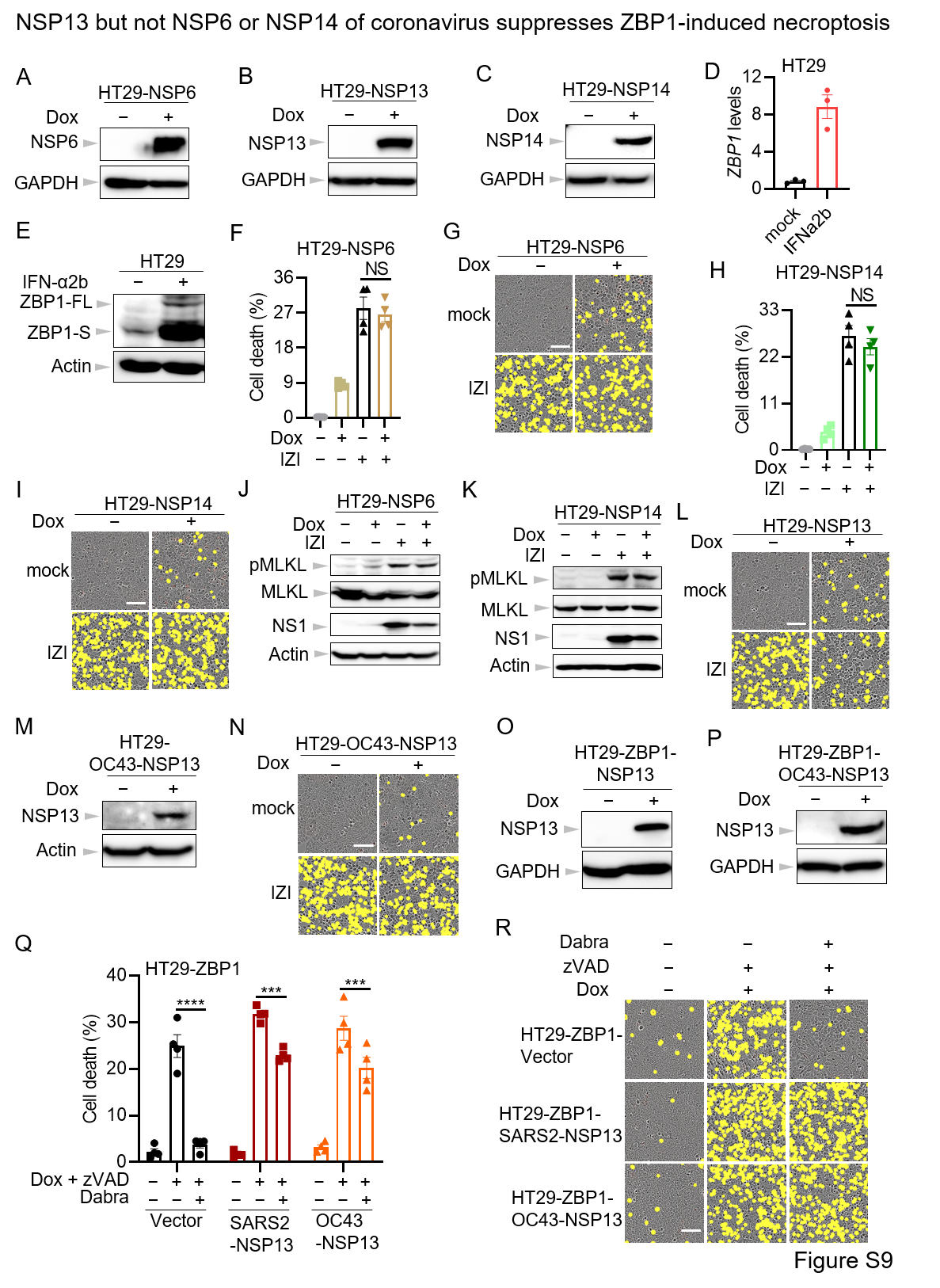


**Figure S9. NSP13 but not NSP6 or NSP14 of coronavirus suppresses ZBP1-induced necroptosis, related to Figure 5.**

(A–C) Immunoblot analysis of SARS-CoV-2 NSP6 (A), NSP13 (B) and NSP14 (C) in HT29-NSP6, HT29-NSP13 and HT29-NSP14 cells in the presence of 500 ng/ml doxycycline (Dox) for 24 h. GAPDH is used as the internal control. (D) Real-time PCR analysis of endogenous ZBP1 mRNA in HT29 cells after stimulation with 100 ng/ml interferon-α2b (IFN-α2b) for 24 h, presented relative to levels of *ACTIN*. (E) Immunoblot analysis of full length ZBP1 (ZBP1-FL) and short isoform of ZBP1 (ZBP1-S) in wild type HT29 cells in the presence of 100 ng/ml IFN-α2b for 24 h. Actin is used as the internal control. (F) Cell death analysis by IncuCyte in HT29-NSP6 cells infected by influenza A virus (IAV) together with zVAD plus IFN-α2b (IZI) in the presence of 500 ng/ml Dox for 20 h. (G) Representative images of cell death in F. The *yellow* in the images denotes the dead cells counted during the analysis. Scale bar, 100 μm. (H) Cell death analysis by IncuCyte in HT29-NSP14 cells treated with IZI in the presence of 500 ng/ml Dox for 20 h. (I) Representative images of cell death in H. The *yellow* in the images denotes the dead cells counted during the analysis. Scale bar, 100 μm. (J and K) Immunoblot analysis of phosphorylated mixed lineage kinase domain-like protein (pMLKL), total MLKL, and influenza NS1 protein in HT29-NSP6 (J) and HT29-NSP14 (K) cells treated with IZI in the presence of 500 ng/ml Dox for 20 h. Actin is used as the internal control. (L) Representative images of cell death in HT29-NSP13 cells treated with IZI in the presence of 500 ng/ml Dox for 20 h. The *yellow* in the images denotes the dead cells counted during the analysis. Scale bar, 100 μm. (M) Immunoblot analysis of hCoV-OC43 NSP13 in HT29-OC43-NSP13 cells in the presence of 500 ng/ml Dox for 24 h. (N) Representative images of cell death in HT29-OC43-NSP13 cells treated with IZI in the presence of 500 ng/ml Dox for 20 h. The *yellow* in the images denotes the dead cells counted during the analysis. Scale bar, 100 μm. (O and P) Immunoblot analysis of SARS-CoV-2 NSP13 in HT29-ZBP1-NSP13 (O) and OC43 NSP13 (P) in HT29-ZBP1-OC43-NSP13 cells in the presence of 500 ng/ml Dox for 24 h. GAPDH is used as the internal control. (Q) Cell death analysis by IncuCyte in HT29-ZBP1-Vector, HT29-ZBP1-NSP13 and HT29-ZBP1-OC43-NSP13 cells treated with Dox plus zVAD in the absence or presence of Dabrafenib (Dabra) for 24 h. (R) Representative images of cell death in Q. The *yellow* in the images denotes the dead cells counted during the analysis. Scale bar, 100 μm. NS, not significant; ****P* < 0.001; *****P* < 0.0001. Analysis was performed using one-way ANOVA (F and H) or two-way ANOVA (Q). Data are shown as mean ± SEM (n = 3) (D) or (n = 4) (F, H, and Q). Data are representative of three independent experiments.


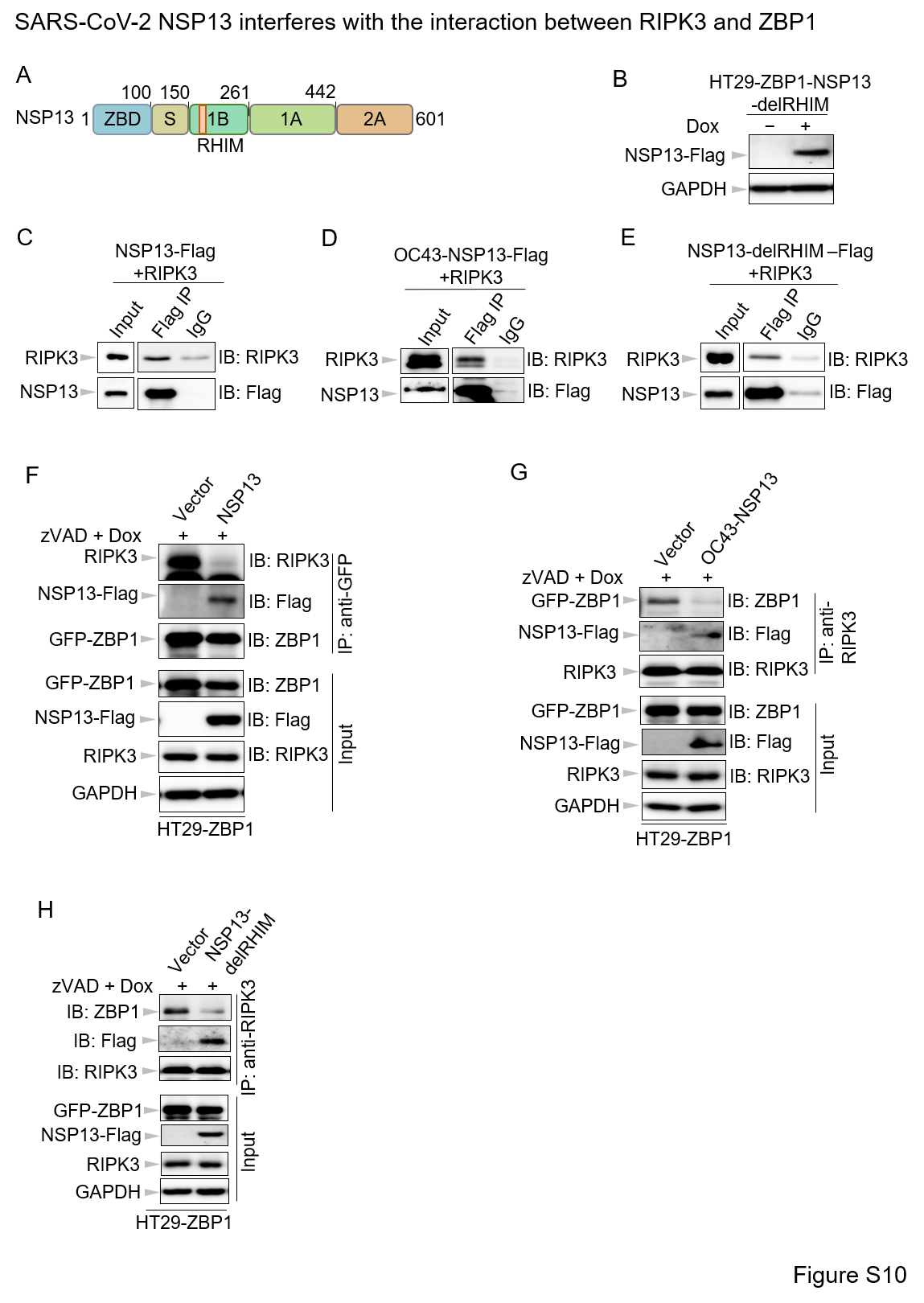


**Figure S10. SARS-CoV-2 NSP13 interferes with the interaction between RIPK3 and ZBP1, related to Figure 5.**

(A) schematic depiction of the domains in SARS-CoV-2 NSP13. ZBD, Zinc binding domain; S, stalk domain; 1B, beta-barrel 1B domain; 1A, RecA like helicase subdomain 1; 2A, RecA like helicase subdomain 2. (B) Immunoblot analysis of NSP13-delRHIM in HT29-ZBP1-NSP13-delRHIM in the presence of 500 ng/ml doxycycline (Dox) for 24 h. GAPDH is used as the internal control. (C−E) Immunoprecipitates and total lysates from HEK293T cells after co-transfection of SARS-CoV-2 NSP13-Flag (C), hCoV-OC43 NSP13-Flag (D), or SARS-CoV-2 NSP13-delRHIM-Flag (E) with RIPK3 for 48h. (F−G) Immunoprecipitates and total lysates from HT29-ZBP1-NSP13 cells (F), HT29-ZBP1-OC43-NSP13 cells (G), and HT29-ZBP1-NSP13-delRHIM (H) in the presence of 500 ng/ml Dox and zVAD for 20 h. Data are representative of three independent experiments.


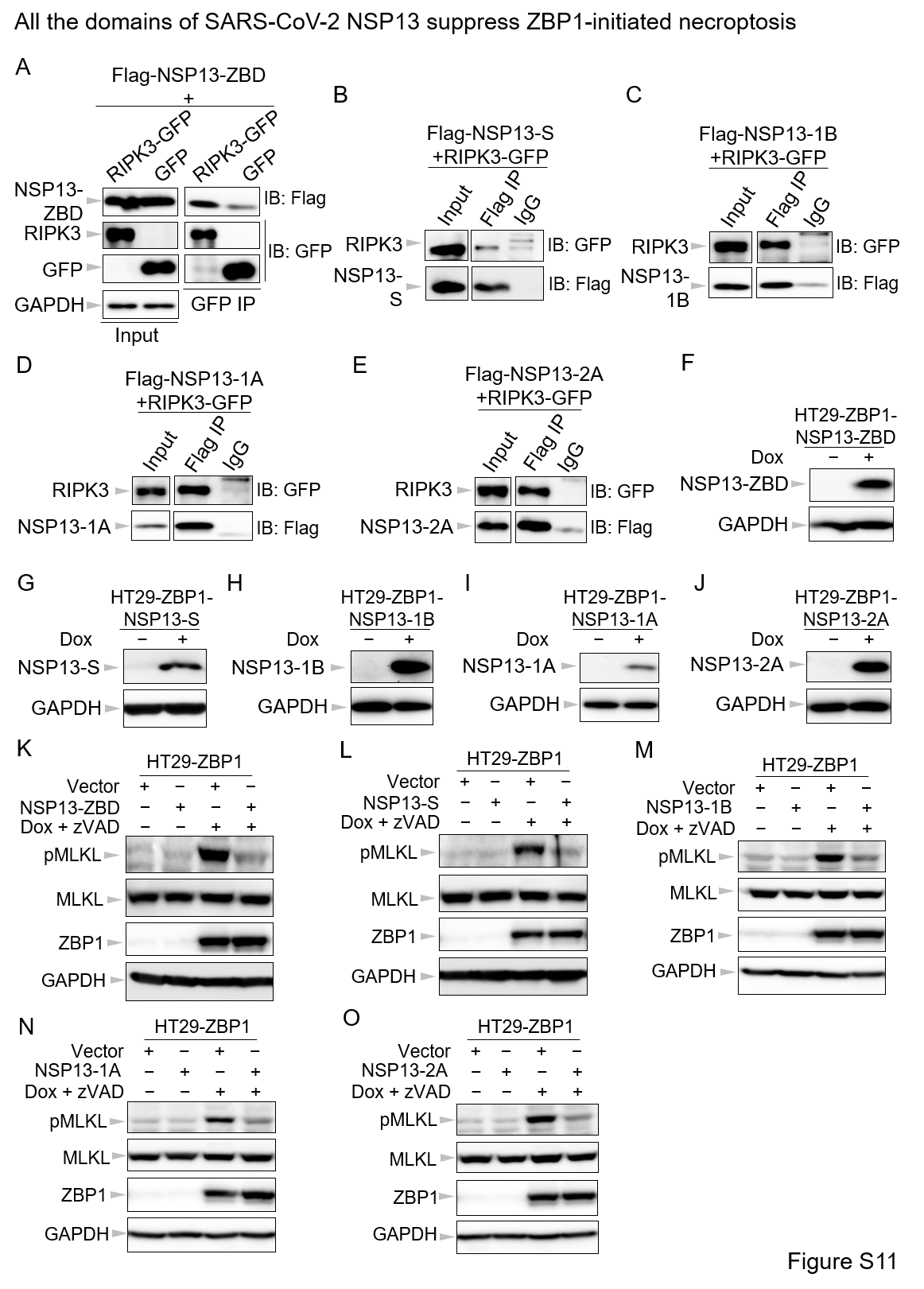


**Figure S11. All the domains of SARS-CoV-2 NSP13 suppress ZBP1-initiated necroptosis, related to Figure 5.**

(A−E) Immunoprecipitates and total lysates from HEK293T cells after co-transfection of NSP13-ZBD (A), NSP13-S (B), NSP13-1B (C), NSP13-1A (D), or NSP13-2A (E) with GFP-tagged RIPK3 for 48h. (F−J) Immunoblot analysis of NSP13 truncation mutants in HT29-ZBP1-NSP13-ZBD cells (F), HT29-ZBP1-NSP13-S cells (G), HT29-ZBP1-NSP13-1B cells (H), HT29-ZBP1-NSP13-1A cells (I), and HT29-ZBP1-NSP13-2A cells (J) in the presence of 500 ng/ml doxycycline (Dox) for 24 h. (K−O) Immunoblot analysis of phosphorylated mixed lineage kinase domain-like protein (pMLKL), total MLKL and ZBP1 in HT29-ZBP1-NSP13-ZBD cells (K), HT29-ZBP1-NSP13-S cells (L), HT29-ZBP1-NSP13-1B cells (M), HT29-ZBP1-NSP13-1A cells (N), and HT29-ZBP1-NSP13-2A cells (O) in the presence of 500 ng/ml doxycycline (Dox) for 20 h. GAPDH is used as the internal control. Data are representative of three independent experiments.


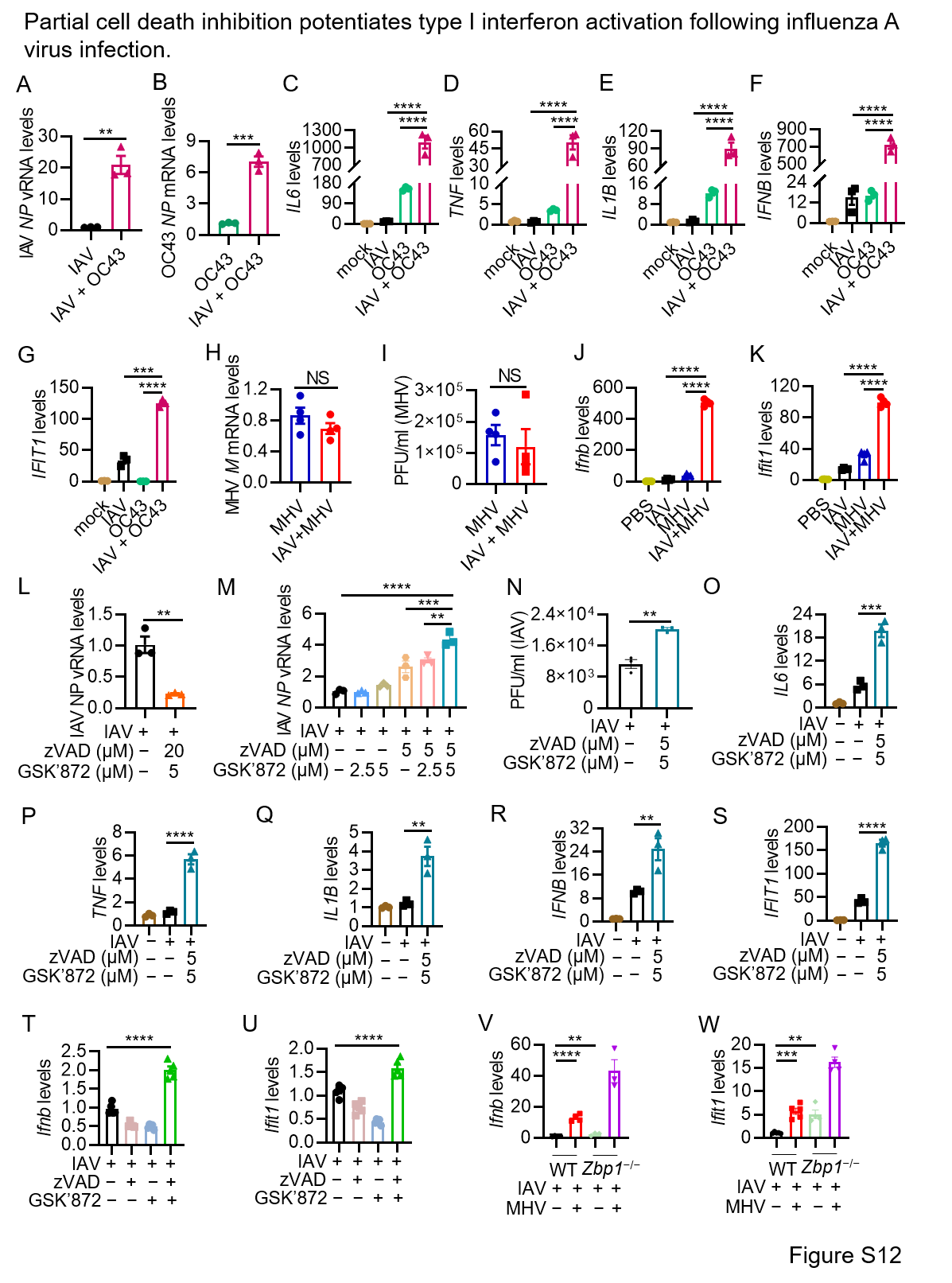


**Figure S12. Partial cell death inhibition potentiates type I interferon activation following influenza A virus infection, related to Figure 6.**

(A−G) Real-time PCR analysis of the expression of influenza A virus (IAV) NP vRNA levels (A), OC43 NP mRNA levels (B), *IL6* (C), *TNF* (D), *IL1B* (E), *IFNB* (F), and *IFIT1* (H) in Beas-2B-ZBP1 cells with the indicated treatment, presented relative to levels of the host gene *ACTIN*. (H) Real-time PCR analysis of the expression of mouse hepatitis virus (MHV) M mRNA levels in the lungs of mice infected with the indicated viruses, presented relative to levels of the host gene *18s rRNA.* (I) Lung MHV titers in mice infected with the indicated viruses. (J and K) Real-time PCR analysis of the expression of *Ifnb* (J) and *Ifit1* (K) in the lungs of mice with the indicated treatment, presented relative to levels of the host gene *18s rRNA.* (L) Real-time PCR analysis of the expression of IAV NP vRNA levels in IAV-infected Beas-2B-ZBP1 cells with or without 20 μM zVAD plus 5 μM GSK’872, presented relative to levels of the host gene *ACTIN*. (M) Real-time PCR analysis of the expression of IAV NP vRNA levels in Beas-2B-ZBP1 cells with the indicated treatment. (N) IAV titers in IAV infected Beas-2B-ZBP1 cells with or without 5 μM zVAD plus 5 μM GSK’872. (O−S) Real-time PCR analysis of the expression of *IL6* (O), *TNF* (P), *IL1B* (Q), *IFNB* (R), and *IFIT1* (S) in IAV infected Beas-2B-ZBP1 cells with or without 5 μM zVAD plus 5 μM GSK’872, presented relative to levels of the host gene *ACTIN*. (T and U) Real-time PCR analysis of the expression of *Ifnb* (T) and *Ifit1* (U) in the lungs of mice with the indicated treatment, presented relative to levels of the host gene *18s rRNA.* (V and W) Real-time PCR analysis of the expression of *Ifnb* (V) and *Ifit1* (W) in the lungs of mice infected with the indicated virus, presented relative to levels of the host gene *18s rRNA.* NS, not significant; ***P* < 0.01, ****P* < 0.001, and *****P* < 0.0001. Analysis was performed using Student’s *t* test (A, B, H, I, L, and N), one-way ANOVA (C–G, J and K, M, and O–U), or two-way ANOVA (V and W). Data are shown as mean ± SEM (n = 3) (A–G and L–S), (n=4) (H–K, T and U), or (n=4–5) (V and W). Data are representative of three independent experiments.
